# Surviving the Extremes: Seasonal Dynamics of Photochemical Performance in Plants From Cold‐Arid Himalayan Mountains

**DOI:** 10.1111/ppl.70269

**Published:** 2025-05-19

**Authors:** Thinles Chondol, Jorge Gago, Jaume Flexas, Javier Gulías, María José Clemente‐Moreno, Jan Binter, Jiří Doležal

**Affiliations:** ^1^ Department of Functional Ecology Institute of Botany, Czech Academy of Sciences Třeboň Czechia; ^2^ Department of Botany, Faculty of Science University of South Bohemia České Budějovice Czechia; ^3^ Research Group on Plant Biology Under Mediterranean Conditions Universitat de les Illes Balears (UIB) – Instituto de Investigaciones Agroambientales y de Economía del Agua (INAGEA) Palma Spain; ^4^ Department of Experimental Plant Biology, Faculty of Science Charles University Prague Czech Republic

**Keywords:** alpine and subnival ecosystems, chlorophyll fluorescence, cold‐arid mountains, *F*_*v*_/*F*_*m*_, Himalayas, leaf traits, photochemical performance of PSII, seasonal dynamics

## Abstract

Plants in extreme environments face pronounced seasonal variations in abiotic conditions, influencing their growth and carbon gain. However, our understanding of how plants in cold‐arid mountains sustain carbon assimilation during short growing seasons remains limited. Here, we investigate seasonal dynamics and interspecific variability in photochemical performance of 310 individuals, comprising 10 different dicotyledon plant species across 3100–5300 m in the NW Himalayas, spanning semi‐deserts to subnival zones. From early June to late September, we measured *F*_*v*_/*F*_*m*_ and ΦPSII, assessing ΦPSII relationships with leaf traits (N, P, C, C:N ratio, LMA, and LDMC) and environmental factors (temperature, soil moisture content, etc.). Our findings revealed that high‐Himalayan plants maintained relatively stable photosynthetic performance (*F*_*v*_/*F*_*m*_ = 0.7–0.85), indicating optimal function even under potential stress. Contrary to our hypothesis that ΦPSII peaks mid‐season in alpine and subnival zones and early season in steppes and semi‐deserts, it declined by 33% across species and habitats throughout the season. This decline was closely associated with nutrient depletion, leaf senescence, and energy–water limitations. Species exhibited distinct strategies, with some prioritising structural resilience over photosynthesis, while others optimised photochemical performance despite environmental constraints. Alpine and subnival plant performance was constrained more by soil moisture deficits and high temperatures than cold temperatures, while deep‐rooted steppe and semi‐desert plants were primarily constrained by high temperatures and evaporative forcing rather than soil moisture deficit. These results provide new insights into how Himalayan plants adapt to extreme environmental conditions, highlighting the crucial interplay between moisture and temperature in shaping their performance within cold‐arid mountains.

## 1 Introduction

Natural environments are characterised by complex abiotic and biotic constraints, which can challenge plant photosynthetic performance through stressors like heat, cold, resource limitations, and seasonal variations. These stressors often interact or occur sequentially during the growing season, impacting plant survival and productivity (Zandalinas and Mittler 2022). However, plants can partially adapt to these challenges as part of their survival strategies (Chaves et al. 2009; Lawlor 2009). Unlike typical alpine zones that are persistently cold and moist, the high‐Himalayan mountains range from hot‐arid, prone to seasonal drought and intense summer heat at lower elevations, to cold‐moist, prone to freezing at the highest alpine zones. In such an environment, plant response is strongly driven by an intricate interaction between various abiotic factors (Han et al. 2023) that co‐limit plant photosynthesis. The growing season in this environment typically lasts from 3 to 4 months to just a few weeks at the highest elevations (Doležal et al. 2016), and the environment is nutrient‐limited, mainly due to low microbial activity restricted by low temperature at higher and moisture shortage at lower elevations (Macek et al. 2012).

Low temperatures and reduced soil moisture significantly impact photosynthesis by decreasing gas exchange due to stomatal closure, mesophyll conductance constraints, and limiting PSII activity, ultimately reducing plant performance (Clemente‐Moreno et al. 2020a, 2020b; Moore et al. 2021; Rojas et al. 2022; Zhai et al. 2024). These effects vary across species, habitats, and seasons, highlighting the need to capture interspecific differences and seasonal nuances in plants from diverse environments. To cope, plants have evolved various adaptive strategies (see Fernández‐Marín et al. 2020). In arid habitats, they develop deep and lateral roots for water access (Doležal et al. 2024), while in cold, high‐altitude regions, small, cushiony alpine plants enhance cold tolerance by decoupling from air temperatures, producing osmoregulatory metabolites, antioxidants, and anti‐stress proteins (Liancourt et al. 2020; Chlumská et al. 2022; Gago et al. 2023).

Most ecophysiological studies focus on temperate forests, moist alpine, or arctic environments (García‐Plazaola et al. 2015; Fernández‐Marín et al. 2020; Gago et al. 2023; Mehta and Chawla 2024), and woody species (Yin et al. 2009; Rathore et al. 2018) or crop plants grown mostly in a controlled setting (Lu and Zhang 1999; Dos Santos et al. 2013; Mesa et al. 2022), leaving the cold‐arid high‐Himalayas largely understudied. Research from grasslands, shrublands, and mixed forest shows that photosynthetic capacity either declines over the growing season (Goedhart et al. 2010) or peaks mid‐season (Burnett et al. 2021). Himalayan studies, such as Rathore et al. (2018) on *Rhododendron anthopogon* and Rahman et al. (2020) on *Pedicularis punctata* and *Plantago major* , examined seasonal leaf traits and cold acclimation via phytohormone concentrations but did not assess photochemical performance or gas exchange. Flexas et al. (2022) and Mehta and Chawla (2024) are among the few studies using in situ photosynthesis measurements across multiple Himalayan species (however, within 1000–4000 m asl), highlighting interspecific variations in stress tolerance and performance. Moreover, much of the research highlights trait variation across different elevations or habitats; there is a lack of emphasis on seasonal changes in plant performance, with only a few studies, such as those by Jurik (1986) and Gast et al. (2020), however, focusing exclusively on woody species. Seasonal studies are critical, as factors like mid‐season heat and drought in arid zones or early/late‐season cold stress at high elevations are expected to influence leaf traits, stress tolerance, and photosynthetic performance (Römermann et al. 2016; Gast et al. 2020). The scarcity of in situ seasonal data reflects the logistical and resource challenges of fieldwork in these remote and extreme environments.

Photosynthetic measurements such as ΦPSII, *F*_*v*_/*F*_*m*_, and gas exchange are often integrated with functional leaf traits like leaf mass area (LMA), leaf dry matter content (LDMC), and leaf nutrient content in ecophysiological studies. This helps to gain deeper insights into plant performance and to identify functional adaptations (Maxwell and Johnson 2000; Bucher et al. 2021; Flexas et al. 2022). Leaf traits, such as dry matter content (LDMC) and leaf mass per area (LMA), are indicative of a plant's resource‐use strategy, often reflecting a trade‐off between rapid carbon assimilation and efficient resource conservation (Pérez‐Ramos et al. 2013; Onoda et al. 2017). These traits are often employed as a proxy for stress tolerance and structural support (Körner and Renhardt 1987; Vile et al. 2005). Environmental conditions influence these traits; for instance, plants produce tougher leaves with higher dry matter content in warmer and drier habitats, enhancing drought tolerance by minimising wilting (Vile et al. 2005). Conversely, leaves are often thinner and more water‐rich in moist, nutrient‐limited environments, facilitating nutrient acquisition (Poorter et al. 2009; Liu et al. 2020).

Nutrients like nitrogen (N), phosphorus (P), and potassium (K) are vital for plant photosynthesis and play a key role in enabling plants to develop stress tolerance mechanisms (see Gago et al. 2023). N is essential in chloroplast components such as Rubisco, photosystem proteins, and chlorophyll. P supports energy metabolism by facilitating ATP and NADPH production and driving the Calvin–Benson Cycle (Köhler et al. 2006; Lambers and Oliveira 2019; De Bang et al. 2021). However, plants often prioritise structural growth and stress tolerance in nutrient‐limited environments over enhancing their photosynthetic performance. While this adaptation may help manage oxidative stress and improve survival under extreme conditions, it often decreases photosynthetic performance (Bucher et al. 2021; Khan et al. 2022; Gago et al. 2023). Water stress, along with the effect of intensive solar exposure, can inhibit photosynthesis by damaging the oxygen‐evolving complexes in PSII and its reaction centers (Lu and Zhang 1999; Zavafer et al. 2015), ultimately leading to a reduction in plant photosynthetic performance (Flexas et al. 2006; Yin et al. 2009; Batra et al. 2014).

Temperature and soil moisture's impact has been extensively discussed in studies such as Zhang et al. (2023), Reyes‐Bahamonde et al. (2022), Flexas et al. (2022), and Reich et al. (2018), highlighting moisture limitation and temperature extremes to reduce stomatal and mesophyll conductance to CO_2_, thereby decreasing N‐use efficiency (Hikosaka 2004) and photosynthetic carbon gain (Letts et al. 2009; Zhang et al. 2023). Limited moisture impairs photobiochemistry, induces oxidative stress, leads to increased antioxidant activity, and lastly, senescence and programmed cell death (Flexas et al. 2006). Furthermore, it also influences microbial N_2_ fixation, organic matter decomposition, and nutrient availability, which are crucial for plant performance in cold ecosystems (Wang et al. 2016; Rousk et al. 2018; Gago et al. 2023).

This study investigates seasonal dynamics and interspecific variations in the photochemical performance of plant species across contrasting habitats and elevations (3100–5300 m) in the cold, arid, high Himalayan mountains. It highlights the influence of leaf traits and environmental factors on plant performance. With the Himalayas experiencing rapid climate change, unprecedented extreme weather events, and intensified land‐use changes (Dvorský et al. 2011), these findings are essential for understanding plant survival strategies in extreme conditions and predicting their responses to seasonal stressors like drought, heat, and cold (Burnett et al. 2021).

**FIGURE 1:**
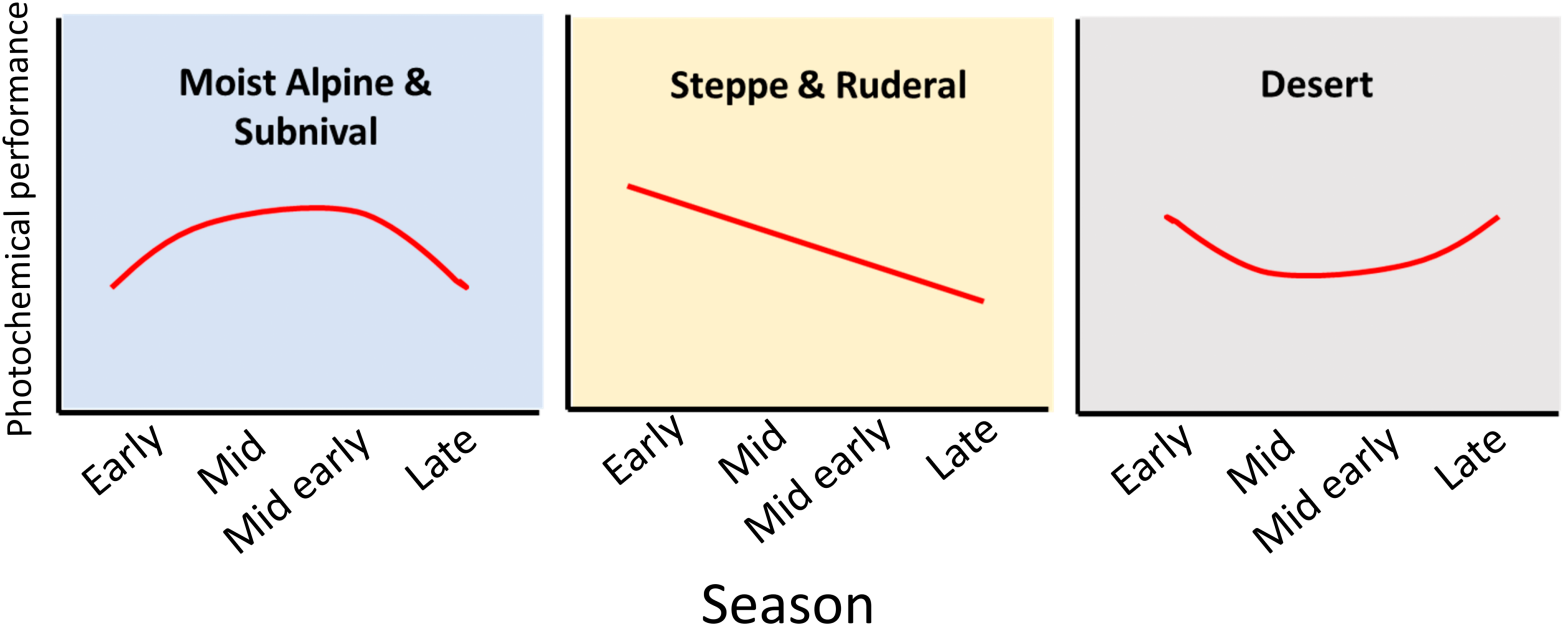
A graphical representation of the expected seasonal pattern of the photochemical performances of the plants from contrasting environments.

Our primary research questions were: (1) How does photochemical performance in high‐Himalayan plants vary throughout the growing season, and are there interspecific differences? We hypothesise that (1a) Plants in moist, cold habitats at higher elevations will likely achieve peak photochemical performance during the mid‐growing season when environmental conditions are most favourable. Conversely, optimal performance is anticipated early in the growing season in dry, hot habitats at lower elevations when conditions are comparatively more suitable despite the overall aridity (Figure 1); (1b) *Waldheimia tridactylites*, which was sampled exclusively at the subnival site, will exhibit the highest photochemical performance due to the short growing season in these environments, where rapid carbon gain is essential for survival during the prolonged winter months. However, interspecific variability is hypothesised to depend on their trade‐off strategy between photosynthesis and structural tolerance.

(2) Which leaf traits and environmental factors influence photochemical performance, and to what extent? We assumed that: (2a) The seasonal variation in leaf nutrient content will mirror the seasonal pattern of plant photochemical performance, as key nutrients such as nitrogen (N) and phosphorus (P) directly impact photosynthesis; (2b) Greater structural investment in leaves will negatively affect photochemical performance, and (2c) Low temperatures will likely limit photochemical performance in moist alpine and subnival habitats, whereas soil moisture will be a primary limiting factor in arid steppe environments.

## 2 Material and Methods

### 2.1 Study Site and Experimental Design

**FIGURE 2:**
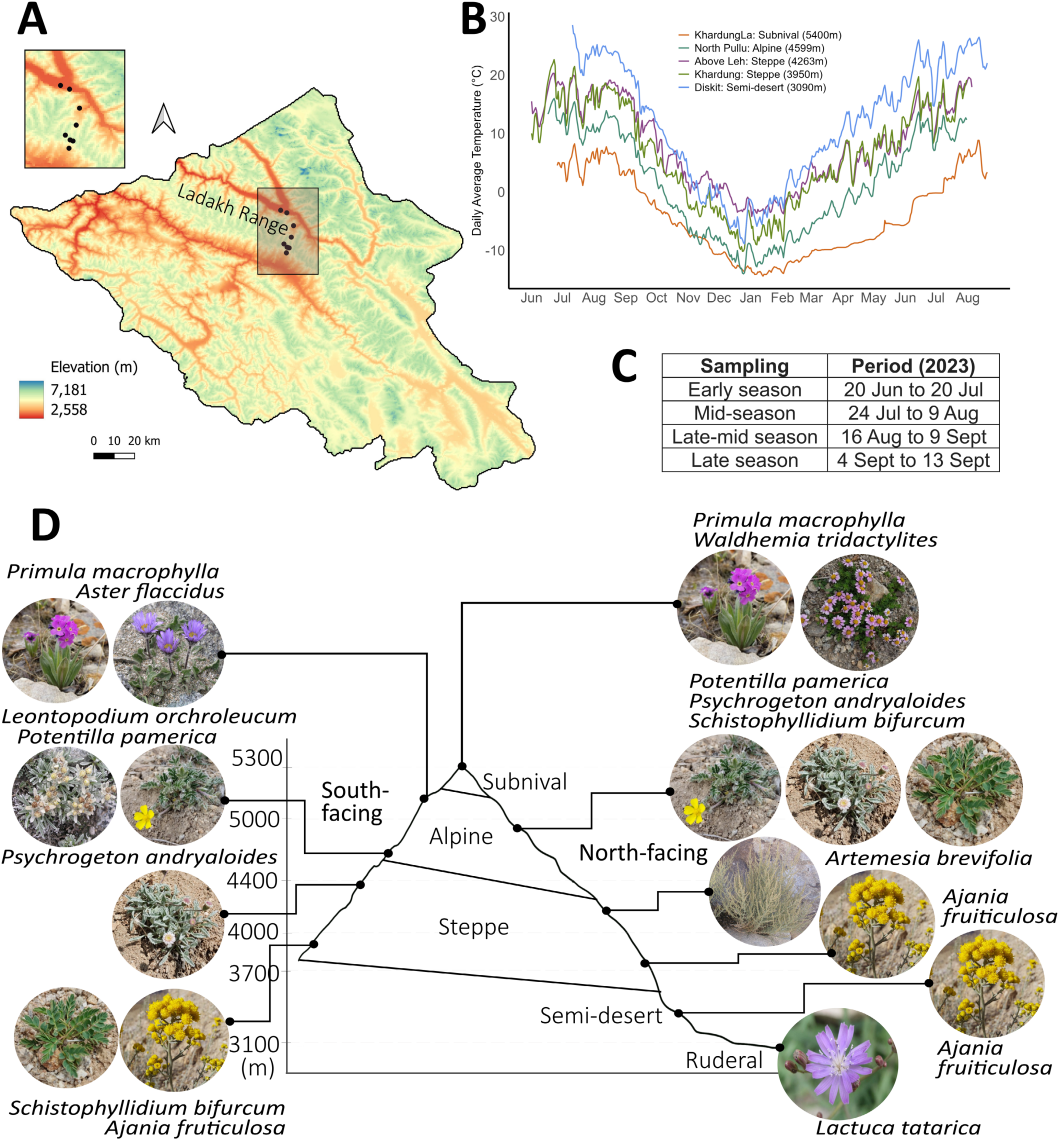
(A) Map of Ladakh, India, showing the study site highlighted in grey and sampling locations as black dots across the Ladakh range (also enlarged in the left corner). (B) Average annual temperatures at −6 cm depth (2022–2023) at sampling locations in different habitats. (C) Table showing the exact dates during which the measurements were made through the growing season categorised into—Early, Mid, Late‐mid, and Late. (D) Experimental design depicting elevations, habitat types, and species measured from each site.

The study was conducted during the growing season (June to September 2023) on dicot plant species in the Ladakh range of the NW Himalayas, India. The study transect extended along an elevation gradient from 4200 to 5300 m above sea level (asl) on the south‐facing slope and down to 3100 m on the north‐facing slope. This gradient encompassed a variety of habitats, including arid steppe, semi‐desert, and productive ruderal zones at lower elevations, moist alpine habitats at higher elevations, and subnival zones near glaciers at the highest elevations (Figure 2).

The region exhibits distinct environmental conditions at different elevations, with significant day‐night and seasonal variability. As recorded by the TMS dataloggers (Wild et al. 2019) installed in 2021 and 2022, the annual mean temperature recorded at −6 cm depth was −5.6°C at the highest site (Figure 2). The absolute maximum temperature recorded at −6 cm under soil was 8.2°C and the absolute minimum was −14.4°C. At the lowest site (3100 m), the annual mean temperature recorded at −6 cm depth was 10.35°C with an absolute maximum of 28.6°C and a minimum of −8.9°C. The growing season varies by elevation, defined by days with a maximum temperature of at least 3°C (Körner and Renhardt 1987). At the highest elevations, it lasts about 2–3 months, while at the lower elevations, it extends for about 4 months, based on the climate data obtained from TMS dataloggers installed across the study site.

In our transect, we selected 10 dominant species, comprised of families such as Asteraceae (7), Rosaceae (2), and Primulaceae (1) (Figures 2 and S1). Some species, such as *Primula macrophylla*, *Potentilla pamerica*, *Psychrogeton andryaloides*, *Schistophyllidium bifurcum*, and *Ajania fruticulosa*, occurred in more than one habitat (Figure 2). All the species were sampled four times during the growing season, except at the highest subnival site, where they were sampled only three times due to the short growing season, with five replicates per species each time, resulting in a total of 310 individuals (details in Table S1). The sampling is categorised into Early, Mid, Late‐mid, and Late growing seasons. Sampling and measurements were conducted from June until September (Figure 2). All measurements were performed on a clear sunny day, and matured, ungrazed, undamaged, and uninfected leaves were selected. Flowering was observed during the first sampling in early June in the lower habitats and during the second sampling in July in the higher habitats. Yellowing of leaves was observed during the third and fourth samplings in late August and September, more prominently in *P. macrophylla* , *S. bifurcum*, and *A. fruticulosa* .

### 2.2 In Situ Photochemical Performance Measurement

We first determined the light‐saturating intensity for each species using light response curves (starting in dark‐adapted conditions) (Table S1). We identified the time required to reach PSII steady‐state conditions under these light‐saturating conditions for discrete measurements (Zhai et al. 2024) by employing 3–4 biological replicates (Table S1). ΦPSII is the effective quantum yield of photosystem II, which tells us about the efficiency with which the light absorbed by chlorophyll is converted through the electron transport chain, contributing to photochemistry (Maxwell and Johnson 2000). For the final measurement of ΦPSII, we randomly selected five individual replicates per species, ensuring they were spaced at least a metre apart to avoid sampling the same individual, particularly for clonal plants. Each selected individual was dark‐adapted for 1 h using a dark opaque cloth and a leaf clip to prevent stray light from entering the leaf (Ritchie 2006; Baker 2008; Kalaji et al. 2014).

Chlorophyll fluorescence was measured using a portable Photosynthetic Yield Analyzer (Mini‐PAM II, Heinz Walz, Effeltrich, Germany). For dark‐adapted individuals, minimal fluorescence (*F*_0_) and maximal fluorescence (*F*_*m*_) were recorded to calculate $F_{v}/F_{m}=\frac{\left(F_{m}-F_{0}\right)}{F_{m}}$, representing the maximum quantum yield of PSII or the efficiency of excitation energy harvested by open PSII reaction centres. For the same individuals, the effective quantum yield of PSII (ΦPSII) was measured (Genty et al. 1989), which is the proportion of light absorbed by chlorophyll molecules in the reaction centre of PSII and used for photochemistry (Maxwell and Johnson 2000).

### 2.3 Leaf Functional Traits Measurement

A few leaves from the same individual were collected, and their fresh weight (FW) was measured in the field instantly using a Kern TGD 50‐3C portable weighing scale (0.001 g precision). The leaves were photographed against a white background with a reference scale bar to analyse for leaf area. A piece of plexiglass was used to hold the leaf flat during imaging (Griffin‐Nolan and Sandel 2023). Later, the acquired images were analysed for leaf area using the open software Fiji ImageJ (Schindelin et al. 2012). Images were analysed using a median filter to reduce noise and Otsu's method of global thresholding algorithm (Otsu 1979).

The leaves used for the trait measurement were consistently from the same individual, although not invariably from the same leaflet where *F*_*v*_/*F*_*m*_ and ΦPSII were measured. The leaves were placed in paper bags and oven‐dried for > 72 h at 60°C until constant weight. The leaf dry matter content (LDMC), which measures the proportion (%) of the dry matter in the leaf (structural investment), was obtained using the formula $\frac{\mathrm{DW}}{\mathrm{FW}}\times 100\left(\mathrm{mg}\;g^{-1}\right)$ (Vaieretti et al. 2007). Undamaged leaves were collected from the same individuals to analyse leaf nutrients such as C, N, and P (~300 mg). The dried leaves were ground to a fine powder using a grinder. Total C and N analysis was performed by combusting at 950°C coupled to individual infrared detection and thermal conductivity (LECO TruspecCN Elemental Analyzer). Total P was measured in an ICP‐OES (Thermo Fisher Series 6500, Thermo Scientific). These analyses were performed at the CEBAS‐CSIC Ionomic Service in Murcia, Spain.

### 2.4 Environmental Variables Measurement

Environmental variables such as the photosynthetically active radiation (PAR, μmol photon m^−2^ s^−1^), air temperature (°C) at +6 cm above the surface, air humidity (%), soil water content (SWC, %), and temperature (°C) at −6 cm depth were measured using a datalogger (MicroLog SDI‐MP, EMS Brno, Czech Republic) which recorded at an interval of 1 min. Long‐term climate data from those sites was obtained from the TMS loggers (Wild et al. 2019) measuring the temperature and soil moisture every 10‐min interval since 2021. A cumulative air temperature was calculated by compiling and analysing temperature data recorded using TMS loggers, measuring every 15 min from the previous year to the specific date on which the measurements were conducted for each site. This was done to make the temperature and soil moisture data comparable and account for the integrated environmental influence on ΦPSII.

### 2.5 Statistical Analysis

To understand how ΦPSII in plants changes throughout the growing season, we employed linear mixed‐effects models (LMM) using the *lmer* function from the *lmerTest* package in R (Kuznetsova et al. 2017). The model was fitted with the day of the year as the independent variable and ΦPSII and *F*_*v*_/*F*_*m*_ as the dependent variables, with species identity included as a random factor. Model summaries and *p* values were obtained using the Type III Analysis of Variance table with Satterthwaite's method. The variance explained by the fixed effect (marginal *R* ^2^) and by both fixed and random effects (conditional *R* ^2^) was calculated using the *r.squaredGLMM* function in the *MuMIn* package (Bartoń 2019). To explore interspecific differences, we conducted multiple comparison tests for each species' ΦPSII and *F*_*v*_/*F*_*m*_ independently using one‐way ANOVA, followed by a post hoc Tukey's test. In most cases, statistical assumptions of normality and homoscedasticity were met; if these assumptions were not met, the Kruskal–Wallis and Bonferroni‐corrected Wilcoxon tests were used. The *MultcompView* package was used to visualise Tukey letters.

To evaluate how leaf functional traits such as nutrient content by mass (%) (N, P, C), C: N, LMA, and LDMC relate to ΦPSII across various species, we fitted a separate linear mixed‐effects model using the *lmer* function from the *lmerTest* package in R (Kuznetsova et al. 2017) for each predictor. The model exploring the influence of leaf traits on ΦPSII used N, P, C, C: N, LMA, and LDMC as fixed effects and species identity as random effects. Similarly, we tested the influence of environmental variables on ΦPSII by fitting a separate mixed‐effect model for each fixed predictor, such as SMC and cumulative air temperature, with species identity included as a random effect. The best model was chosen based on the AIC values after testing models with and without log‐transformations of the independent variables. We fitted a linear model for each species to explore interspecific differences and how the relationships vary across species. All analyses were conducted using R version 4.4.2.

Finally, we conducted a principal component analysis (PCA) using CANOCO 5 software to reduce the dimensionality of our dataset and identify patterns of the influence of leaf traits and environmental variables on ΦPSII across different species and seasonality (ter Braak and Smilauer 2012). In the PCA, all the leaf functional traits and environmental traits are used as response variables, and species × season is used as a supplementary variable. The response variables were log‐transformed (*y* + 1) with centring and standardisation of rows by a norm.

## 3 Results

### 3.1 Seasonal Dynamics and Interspecific Differences in Photochemical Performance

Across all the species, ΦPSII significantly decreased over the growing season (Figure S2), with an overall 33.3% decline. On average, ΦPSII dropped from 0.12 ± 0.04 during the early season to 0.08 ± 0.04 in the late season. The variation in ΦPSII explained by the season (fixed effect) alone was low (*R* ^2^_*m*_ = 9.6%), but when both season and species identity (random effect) were considered, the explained variance increased significantly (*R* ^2^_*c*_ = 68%), indicating a strong species‐specific response (Table 1).

**TABLE 1:** Summary statistics of the linear mixed‐effect model testing the effect of season on ΦPSII and *F*_*v*_/*F*_*m*_ (the model included species identity as a random effect).

	MSS	NumDf	DenDF	*F*	*p*	*R* ^2^_*m*_ (%)	*R* ^2^_*c*_ (%)
ΦPSII	0.05149	1	299.73	91	< 0.001	9.6	68
*F*_*v*_/*F*_*m*_	0.032317	1	304.79	18.82	< 0.001	5.3	16

ΦPSII varied greatly among species, with up to a threefold difference, while no habitat‐related pattern in ΦPSII was observed. The highest ΦPSII was found in alpine plants *P. pamirica* (0.16 ± 0.03) and *P. macrophylla* (0.13 ± 0.03), followed by steppe species *A. fruticulosa* (0.11 ± 0.04), ruderal *Lactuca tatarica* (0.11 ± 0.03), steppe species *P. andryaloides* (0.09 ± 0.03), *Aster flaccidus* (0.09 ± 0.02) from alpine, *Artemisia brevifolia* (0.08 ± 0.01) from the steppe, *Waldheimia tridactylites* (0.07 ± 0.02) from subnival, *Leontopodium ochroleucum* (0.06 ± 0.03) from alpine, and *S. bifurcum* (0.06 ± 0.01) from steppe, showing the lowest ΦPSII (Table S1).

**FIGURE 3:**
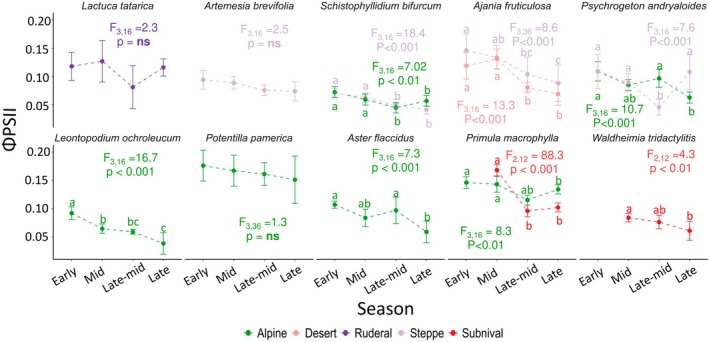
Mean ΦPSII with standard deviation for each species in different habitats across sampling periods. One‐way ANOVA was independently performed per species from various habitats after verifying the normality and homoscedasticity. Letters indicate significant mean differences (Tukey post hoc test).

We did not observe any habitat‐specific seasonal pattern as we hypothesised. All measured species, irrespective of their habitat, showed a significant decline in ΦPSII across the growing season, except for *P. andryaloides* (arid steppe), which showed its lowest ΦPSII mid‐season, in congruence with our hypothesis for species from arid environments (Figure 3). Additionally, *L. tatarica* (ruderal) showed an overall declining trend, as hypothesised, but with an unexpected drop in ΦPSII during the mid‐growing season.

The percentage decline in ΦPSII throughout the season also varied among species and had no habitat‐specific pattern. *L. ochroleucum* experienced the highest seasonal decline (58%), followed by *A. flaccidus* (45%), *A. fruticulosa* (40%), *S. bifurcum* (32%), *W. tridactylites* (27%), *P. andryaloides* (22%), *A. brevifolia* (22%), *P. macrophylla* (19%), *P. pamerica* (15%), and *L. tatarica* (2%).

*F*_*v*_/*F*_*m*_ also decreased, but only by 2.5%, with the mean *F*_*v*_/*F*_*m*_ consistently ranging between 0.85 and 0.75, indicating that these plants were not under severe stress during the growing season (Figure S3). However, a few individuals of *A. fruticulosa* , *S. bifurcum*, *L. ochroleucum*, *P. pamirica* , and *A. flaccidus* showed *F*_*v*_/*F*_*m*_ values below 0.7 at specific times (Figure S3). Seasonality (day of the year) explained 5.3% of the differences in *F*_*v*_/*F*_*m*_ (*R* ^2^_*m*_ = 5.3%), while considering both seasonality and species identity together slightly increased the explained variance to 16% (Table 1). However, species identity alone did not significantly explain the differences in *F*_*v*_/*F*_*m*_.

### 3.2 Leaf Traits

**FIGURE 4:**
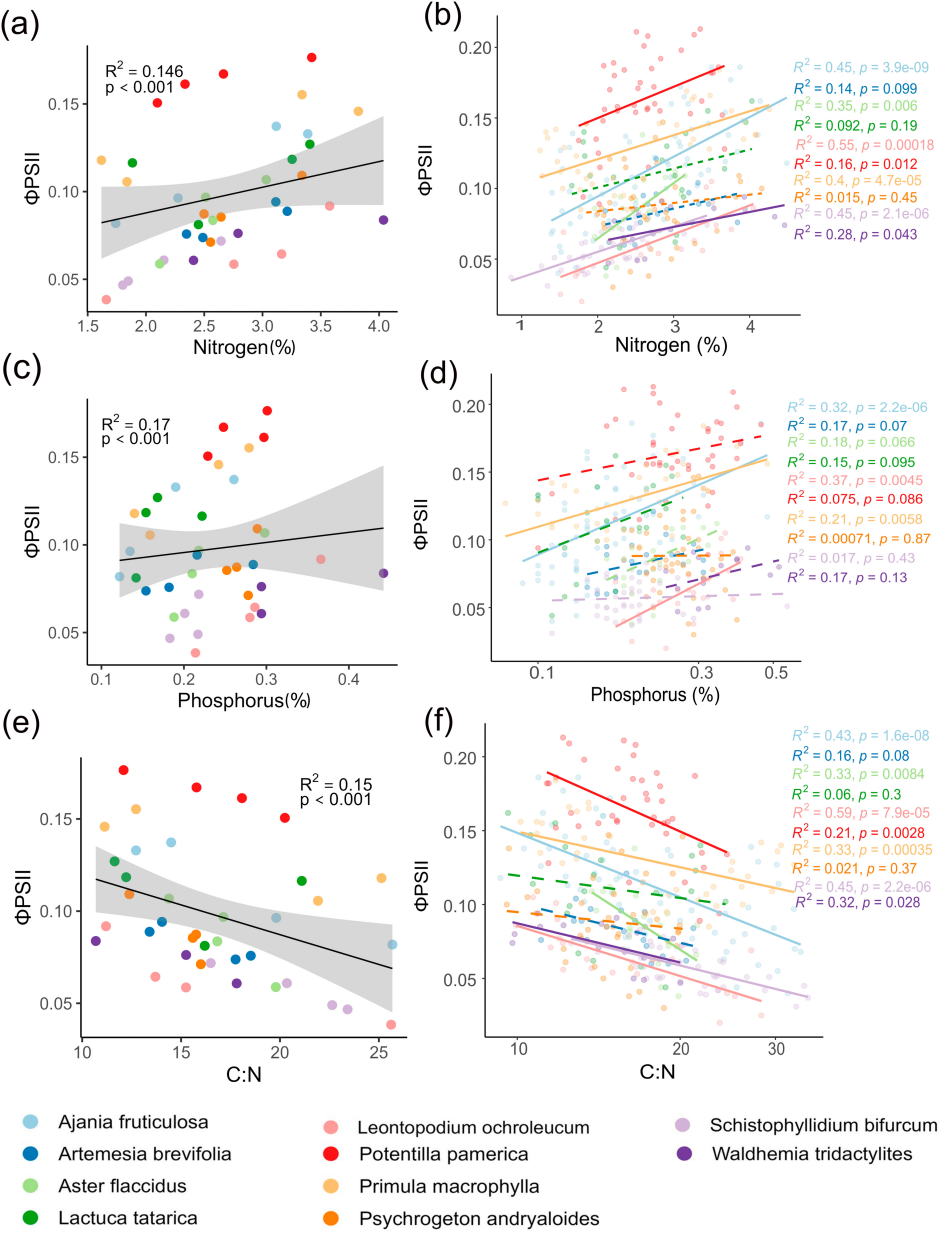
Relationship between ΦPSII and leaf nutrients, and leaf structural traits. Each line with grey areas (95% CI) represents the model‐fitted line to the data. In (A), (C), and (E), points signify species means in each sampling period, while in (B), (D), and (F), points represent individual plants. Different colours in the figure correspond to individual species.

Leaf nutrient profiles exhibited significant changes throughout the growing season. Leaf N levels decreased significantly from 3.21% ± 0.5% at the start to 2.01% ± 0.5% by the end of the season, a 37.38% reduction. Leaf P content dropped from 0.26% ± 0.08% to 0.19% ± 0.08%, a 26.92% decrease. In contrast, leaf C content remained almost constant across species (~42.7%) throughout the season, while the C:N ratio increased across the growing season from 13.6 ± 2.4 to 22.4 ± 5.6 (64.7% increase). A significant interspecific difference in leaf nutrient content was observed, with great variability in the seasonal nutrient decline (Figures S4–S7). *W. tridactylites* (3.08% ± 0.76%) had the highest N, while *S. bifurcum* (2.1% ± 0.5%) had the lowest. *W. tridactylites* also had the highest P content (0.34% ± 0.08%) and *L. tatarica* had the lowest (0.17% ± 0.05%). *A. fruticulosa* had the highest C (44.5% ± 1.63%), whereas *L. tatarica* had the lowest (39.7% ± 1.05%) (Table S1). Additionally, some species had a significant seasonal change in leaf structural traits, such as LDMC and LMA (Figures S8 and S9). However, the changes were not particularly pronounced. Foliar N and P showed a positive association with ΦPSII (Figure 4a,c), while the C:N ratio correlated negatively (Figure 4e). No significant relationships were found between LDMC or LMA and ΦPSII when all species were considered together, though model estimates were negative (*β*_LDMC_ = −0.0008292; *β*_LMA_ = −0.000186) (Figure S10). Linear models for individual species revealed interspecific variation in trait influences on ΦPSII, aligning with overall species trends (Figures 4b,d,f and S10).

### 3.3 Environmental Conditions

**FIGURE 5:**
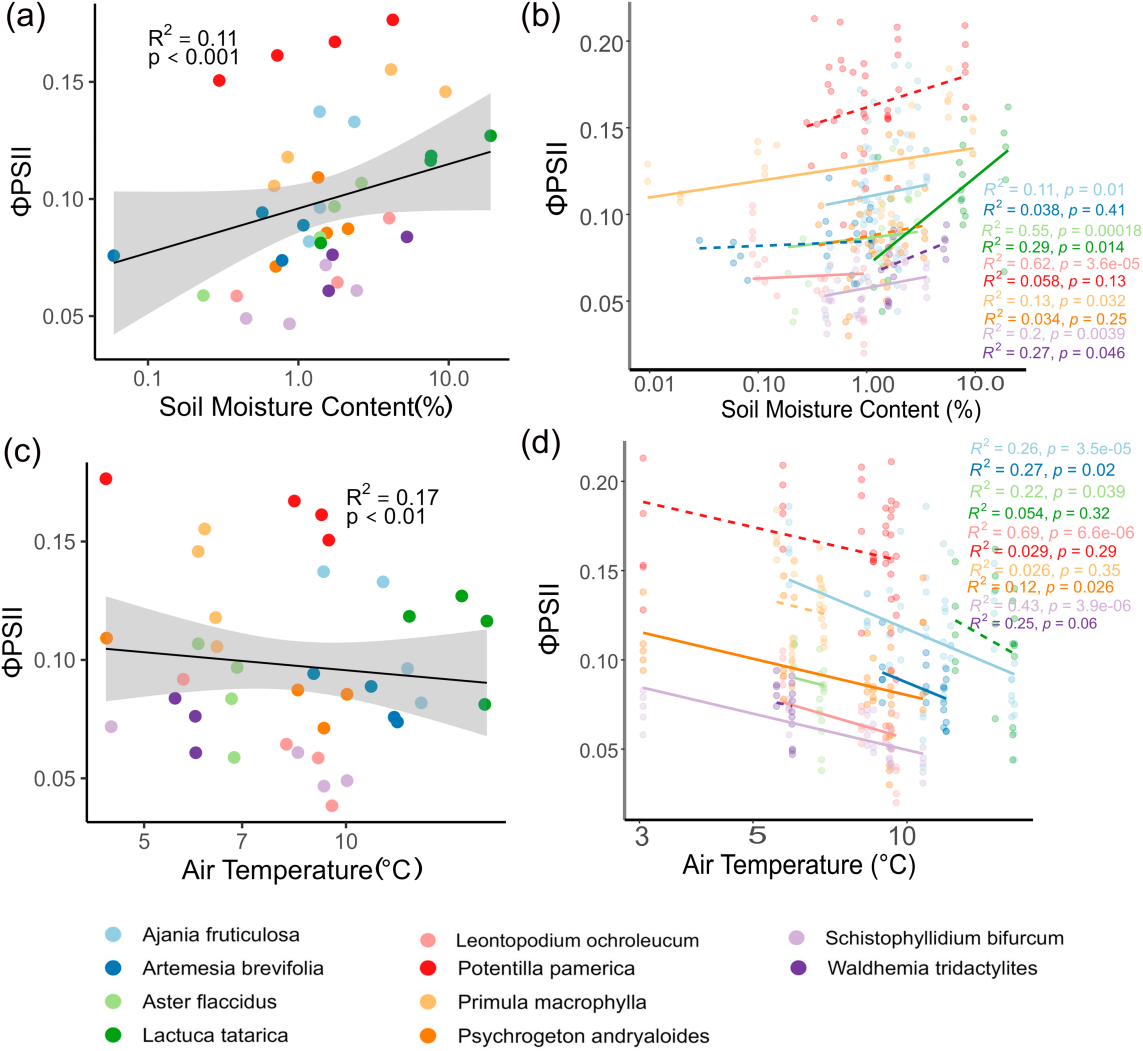
The relationship between ΦPSII and environmental traits: (A) Soil moisture content; (B) Interspecific differences in the influence of SMC; (C) Cumulative air temperature; (D) Interspecific differences in the influence of air temperature. Each line with grey areas (95% CI) represents the model‐fitted line. In (A) and (C), points signify species means in each sampling period, while in (B) and (D), points represent individual plants. Different colours represent different species, with solid lines indicating significant relationships and dashed lines indicating non‐significant relationships.

During the growing season, air temperature and SMC varied significantly across species. Some species experience peak mid‐season temperatures, while others show gradual declines or intermittent fluctuations (Figure S11). Similarly, soil moisture patterns differ, with some species experiencing steady decreases, others peaking mid‐season, and others showing fluctuations throughout (Figure S12). A linear mixed‐effect model analysing all species together revealed that SMC had a significant positive effect on ΦPSII across species (Figure 5a), whereas temperature had a significant negative effect (Figure 5c).

However, when considering each species independently using a linear model, SMC significantly influenced the ΦPSII of some alpine and subnival species, such as *P. macrophylla* , *A. flaccidus* , and *L. orchroleucum*, and some steppe species, such as *A. fruticulosa* , *S. bifurcum*, and ruderal species *L. tatarica* (Figure 5b). Meanwhile, air temperature significantly affected the ΦPSII of species from arid habitats, such as *A. fruticulosa* , *A. brevifolia* , *P. andryaloides*, and *S. bifurcum*, and some species from alpine habitats, such as *A. flaccidus* and *L. orchroleucum* (Figure 5d). For our analysis, we used the average air temperature from the beginning of the growing season to the day of measurement. Steady‐state quantum yield (ΦPSII) has been shown to integrate long‐term acclimatory responses to environmental conditions, which is a suitable measure for assessing ΦPSII over time (Genty et al. 1989; Maxwell and Johnson 2000; Baker 2008).

We found that *A. brevifolia* and *P. andryaloides* from arid steppes are significantly influenced by air temperature, whereas *P. macrophylla* and *W. tridactylites* from moist alpine and subnival areas are constrained by SMC (Figure 5). The ruderal species *L. tartarica* is also influenced by SMC. However, some species, such as *A. fruticulosa* , *S. bifurcum* from the steppe and *L. orchroleucum*, *A. flaccidus* from the alpine, are limited by both air temperature and SMC.

**FIGURE 6:**
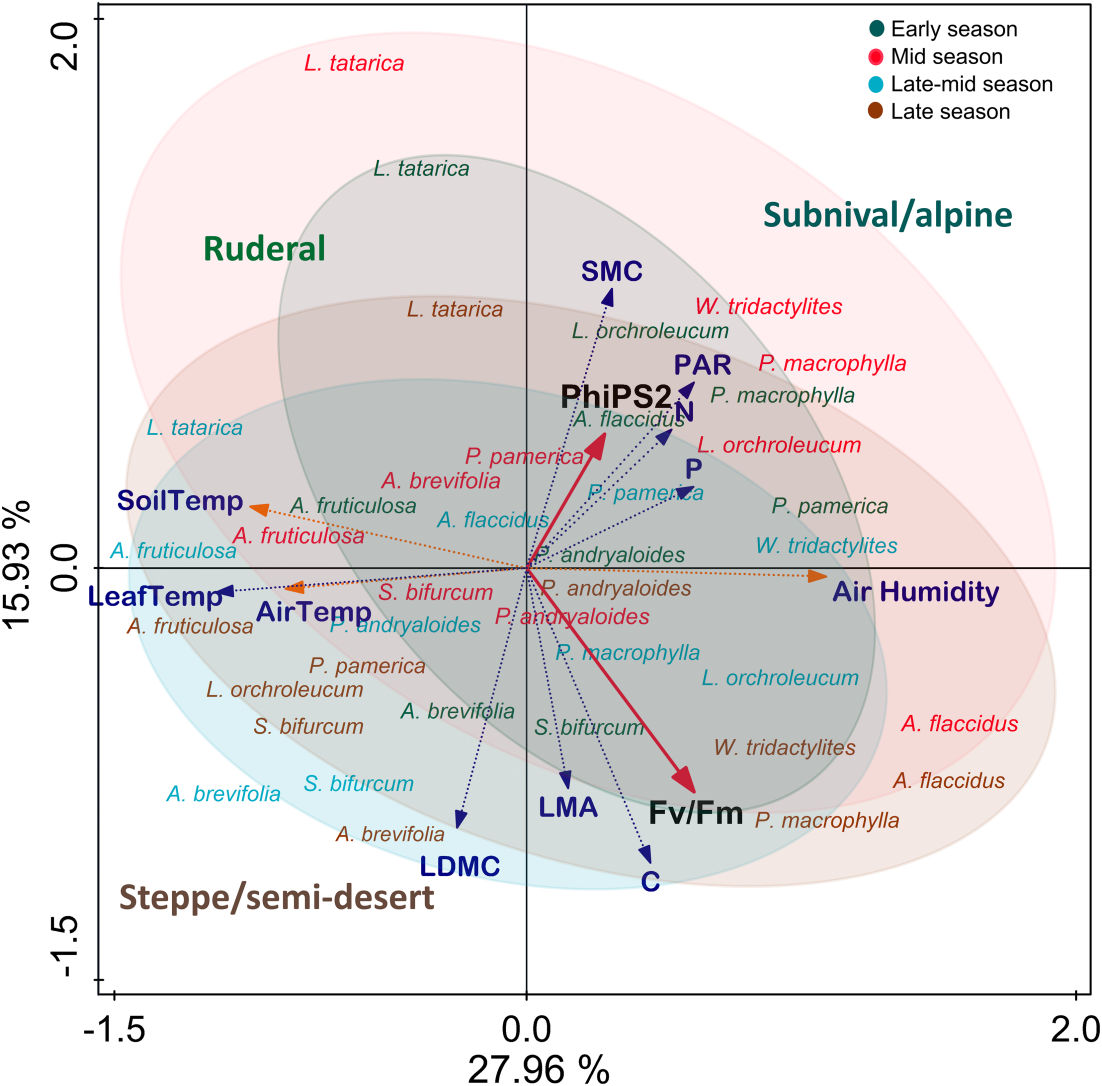
PCA plot examining the relationship between photochemistry, leaf traits (structural and nutrient content), and environmental traits. Variables include LDMC (Leaf Dry Matter Content), LMA (Leaf Mass Area), PAR (Photosynthetically Active Radiation), SoilTemp (soil temperature) –6 cm belowground, AirTemp (air temperature) +6 cm above ground, LeafTemp (leaf temperature), N (Nitrogen), P (Phosphorus), and C (Carbon). Species text colours and ellipses indicate species × season: Green = Early season; Red = Mid‐season; Blue = Late‐mid season; and Brown = Late season.

Furthermore, a multivariate analysis with the leaf traits of all 310 individuals (10 different species) and the environmental variables further aligns with the results obtained from our regression analyses. The ΦPSII of these high‐Himalayan plants during their growing season is more limited by soil moisture deficit and seasonal heat/drought than by low temperature (Figure 6). The first two principal components (PC1 and PC2) explain 27.96% and 15.93% of the total variance, respectively (Figure 6). PC1 is strongly driven by traits like N, P, SMC, and PAR, grouped with the ΦPSII, linking their strong influence on plant photochemistry. The PCA shows that the seasonal dynamics of ΦPSII are highly interspecific and environmentally driven. Those species growing in moist environments (subnival/alpine) were more limited by soil moisture deficit, despite their moist habitat nature, while species from arid habitats were more limited by high temperatures (Figure 6). Our multivariate analysis suggests that temperature and moisture play critical but distinct roles in determining ΦPSII in each plant. Moreover, the intensity of one of them could have synergistic effects in the influence of the other on the plant's physiology.

## 4 Discussion

This study investigated the seasonal dynamics and interspecific variability in the photochemical performance of 310 individuals comprising 10 dicot plant species from elevations ranging from 3100 to 5300 m asl and contrasting habitats (semi‐desert, steppe, alpine, and subnival) within the Ladakh range of the NW Indian Himalayas. In situ measurements of ΦPSII activity were paired with leaf traits, including N, P, and C content, C:N ratio, LMA, LDMC, and environmental factors such as air temperature and SMC. These measurements were conducted four times throughout the growing season (except for subnival sites), with five replicates per species each time. Meanwhile, temperature and SMC were recorded near the sampling sites. Our study demonstrates that high‐Himalayan plants generally maintain optimal functioning, although their photochemical performance declines throughout the growing season. Notably, there is considerable species‐specific variability in ΦPSII activity, reflecting differences in environmental adaptability. We found that ΦPSII is influenced by leaf traits (such as nitrogen, phosphorus, and C:N ratio) and environmental factors (including air temperature and soil moisture). The interspecific variation in the relationship between ΦPSII and leaf nutrient concentrations highlights that each species adopts a distinct ecological strategy, utilising different elements in varying proportions. We found that species from dry habitats are primarily constrained by high temperatures while being low‐moisture adapted. In contrast, species from moist habitats are limited by low available soil moisture; yet, they are well adapted to cold conditions. The study underscores the complex mechanisms governing the photochemical performance of high‐elevation plants, which is chiefly interspecific.

### 4.1 Seasonal and Interspecific Variations in Plant Photochemical Performance

We discovered that the high‐Himalayan plants appeared to function optimally, with *F*_*v*_/*F*_*m*_ values consistently ranging from 0.7 to 0.85, even under potentially stressful conditions, despite some individuals displaying low ΦPSII (Figure S3). This finding aligns with Flexas et al. (2022), suggesting that low ΦPSII in these plants may not necessarily indicate stress but instead reflect an adaptive strategy. Plants with lower photosynthetic rates may be more stress‐tolerant, enabling them to survive during seasonal cold or drought events while sustaining C assimilation, which is probably a strategic trade‐off. We assume that the low *F*_*v*_/*F*_*m*_ at the start of the growing season is likely due to the slow acclimation to the environmental conditions after winter dormancy. In contrast, a low *F*_*v*_/*F*_*m*_ at the end of the growing season in some individuals in our dataset possibly indicates the influence of the onset of senescence (Figure S3). However, the *F*_*v*_/*F*_*m*_ fluctuation likely indicates the difference in adaptability to specific seasonal challenges, such as in *A. flaccidus* and *P. macrophylla* , despite growing in the same habitat.

We observed a seasonal decline in ΦPSII in the studied species, irrespective of the habitat (Figures 3 and S2), which does not entirely follow our Hypothesis 1a (Figure 1), except for *P. andryaloides* (steppe). However, the results were consistent with previous findings on crop plants (Li et al. 2020), temperate grasses (Tejera‐Nieves et al. 2023), woody species (Dos Santos et al. 2013; Marino et al. 2018), and herbaceous plants outside alpine regions (Wada et al. 2023). This decline in ΦPSII arises from a complex interplay of changes in factors such as autumnal leaf yellowing (senescence) (Römermann et al. 2016; Palm et al. 2022), nutrient resorption to perennial belowground organs for winter survival (Zong et al. 2020; Palm et al. 2022; Chondol et al. 2024), reduced SMC (Reich et al. 2018; Tejera‐Nieves et al. 2023), and degradation of chlorophyll compounds and light‐harvesting pigments within the photosynthetic system (Moy et al. 2015). The observed decline in ΦPSII aligns with our findings of seasonal decreases in leaf nutrient content (Figures S4–S7), which are crucial for plant photochemical performance (Fernández‐Marín et al. 2020; Gago et al. 2023). We did not observe a clear mid‐season peak in ΦPSII in moist alpine and subnival plants, nor high ΦPSII values at the start or end of the growing season in dry steppe plants, as initially hypothesised (Figure 1, Hypothesis 1). We suggest this may be because these plants produce new leaves each year, with young leaves being more metabolically active, while mature leaves gradually become slower and less active (Jurik 1986; Wujeska‐Klause et al. 2019). In contrast, the seasonal pattern of the steppe species *P. andryaloides* supports our first hypothesis, potentially due to an abiotic limiting factor that is lowering its ΦPSII during the mid‐growing season.

Despite the general decline in ΦPSII throughout the growing season, we noted significant interspecific differences, highlighting the intricate species‐specific nature of photosynthesis and their adaptability to the environment as hypothesised (1b) (Cai et al. 2007; Gago et al. 2019). For instance, *P. pamerica* seems to be a resilient and efficient species in a resource‐limited environment, showing the highest ΦPSII. *S. bifurcum*, *A. brevifolia* , and *L. ochroleucum* tend to compromise their photochemical performance by using their resources to build tolerant and tough leaves (high dry matter content). *W. tridactylites*, on the other hand, despite having high leaf N content, showed low ΦPSII, contrary to our Hypothesis 1b, and maintained a high carbon content, suggesting a resource‐conservative strategy (Table S1).

### 4.2 Influence of Leaf Traits

Our results showed that ΦPSII is significantly affected by the content of essential elements such as N, P, and C:N ratio in leaves (Figure 4, Hypothesis 2a). The concentration of these nutrients in leaves plays a pivotal role in determining plant photochemical performance, evident from the seasonal decline in ΦPSII, which correlates with the reduced levels of foliar nutrient content. N is particularly crucial for plant photosynthetic machinery as membrane proteins in the electron transport chain, Rubisco in chloroplasts, and the porphyrin ring of chlorophyll molecules (Shi et al. 2006; Hawkesford et al. 2012). A reduction in N can also impair chlorophyll biosynthesis (De Bang et al. 2021).

We also observed a gradual decline in seasonal nutrient levels for most species, thus demonstrating their link with photochemical performance (Figures S4 and S5). This pattern can be attributed to the accumulation of more nutrients to meet the growth requirements at the beginning of the growing season (Liu and Wang 2021), reduced nutrient uptake due to low moisture availability later in the growing season (Macek et al. 2012), or reallocation of nutrients from senescing leaves to other organs (Aerts 1996). Consequently, this decline influences photochemical performance. Interestingly, some species displayed peak nutrient levels in the mid‐growing season, yet no corresponding peak in ΦPSII. This inconsistency may be attributed to heat or drought stress, as this pattern appeared only in plants from drier habitats.

The overall consistent nature of C in the studied species is likely due to its structural role as a key component of cell walls, which are typically less dynamic than other cellular elements (Onoda et al. 2017). However, we did not explore the seasonal dynamics of various non‐structural carbohydrates in this study. Conversely, some species had high C content at the beginning, likely due to the high metabolic activity of the young leaves. Species maintaining a consistent C balance also demonstrate a strong acclimation, allowing them to sustain positive carbon levels under typical conditions while preparing for dormancy as adverse weather approaches (Flexas et al. 2006). Seasonal dynamics and interspecific differences in the concentration of different types of non‐structural carbohydrates (NSCs) across plant organs have been documented in several studies such as Reyes‐Bahamonde et al. (2022), Guo et al. (2025), and Chlumská et al. (2022). These dynamics will likely influence plant photochemical performance, even if total carbon content remains unchanged. However, in the present study, we did not differentiate among various NSC types, which limit our ability to assess their specific functions. Furthermore, the seasonal increase in the C:N ratio is possibly due to the remobilisation of nutrients in perennating organs as a process of leaf ageing (Gago et al. 2023); hence, the negative correlation with ΦPSII.

The interspecific differences we observed in leaf nutrient content and their corresponding ΦPSII values align with Peñuelas et al. (2019), highlighting that each species employs distinct ecological strategies by utilising different elements at varying concentrations. For example, *L. orchroleucum* and *S. bifurcum* exhibited low nutrient content, associated with low ΦPSII and C content. Conversely, *W. tridactylites*, despite possessing high nitrogen content, exhibited low ΦPSII and consistently high C (Table S1). This pattern highlights that high nitrogen content in *W. tridactylites* does not translate into enhanced photosynthetic performance, possibly due to low assimilation rate (conservative strategy) or allocation of N in soluble protein (RuBP) rather than thylakoid proteins (Evans 1989). Alternatively, it can be due to the presence of N in their cell wall components and reduced allocation in photosynthetic proteins (Evans 1989; Onoda et al. 2017). However, in‐depth research can be undertaken to confirm this.

Our regression analysis showed an insignificant influence of LMA and LDMC on ΦPSII (Figure S10). However, the multivariate analysis shows a negative association of investment in leaf structural traits on plant performance, as expected (Hypothesis 2b), which indicates a trade‐off in resource utilisation, prioritising their resource investment in the structural growth of leaves (high LDMC), compromising their photochemical performances (Table S1) (Niinemets 2001; Pérez‐Ramos et al. 2013; Onoda et al. 2017). But species‐specific differences are worth noticing. Most species showed high LMA and LDMC but low ΦPSII, indicating a conservative resource‐use strategy, prioritising structural growth and being highly stress‐tolerant but at the cost of lower photosynthetic efficiency (Niinemets 2001; Hilty et al. 2021; Khan et al. 2022). Our findings align partially with the leaf economic spectrum, where plants at one end focus on productivity, while those at the other end emphasise endurance to maintain a positive carbon balance (Flexas et al. 2006, 2022; Poorter and Bongers 2006; Reich 2014). *L. orchroleucum* showed high LDMC and LMA but also high ΦPSII, which suggests an ability to optimise photosynthetic performance while maintaining structural resilience. Such species might have some additional adaptations, which require more in‐depth studies. On the other hand, *W. tridactylites* and *S. bifurcum* showed a resource‐conservative, low‐performance strategy, possibly due to environmental constraints, indicating a focus on survival strategy. Species like *A. brevifolia* and *L. orchroleucum* prioritise resilience by developing thinner (low LMA) but denser leaves (high LDMC). These species can sustain photosynthetic performance even under stress from drought, nutrient limitations, etc. However, the significant interspecific differences suggest a need for further detailed research, as some species may diverge from this spectrum.

### 4.3 Influence of Environmental Variables

We found that ΦPSII was significantly limited by soil moisture in moist habitats and temperature in arid habitats (Figure 5a,c), contrary to our Hypothesis 2c. However, substantial interspecific variation exists in how temperature and moisture constrain ΦPSII (Figure 5b,d). Species like *A. fruticulosa* and *S. bifurcum* from arid steppes and semi‐deserts face strong co‐limitation due to shallow root systems, leading to drought stress and moisture loss from evaporative transpiration. In contrast, *L. orchroleucum* and *A. flaccidus* from moist habitats are co‐limited by temperature and SMC, with freezing temperatures reducing moisture availability. *A. brevifolia* , while constrained by temperature, benefits from deep roots that reduce moisture limitations. *P. andryaloides*, adapted to arid soils, shows a weak but significant relationship with temperature due to increased evapotranspiration and water loss. Species from moist alpine habitats, such as *A. flaccidus* and *L. orchroleucum*, are sensitive to temperature increases, which affect their access to snowmelt water. However, the significant influence of soil moisture suggests that freezing soils and moisture limitations remain critical. *P. macrophylla* , an alpine forb, thrives in snow‐bed areas and wet springs, relying heavily on soil moisture, which becomes a limiting factor with rising temperatures. *L. tatarica* , adapted to ruderal sites at lower, drier elevations, faces more severe constraints from SMC than temperature. Overall, while some species are well adapted to cold and arid conditions, extreme weather events such as seasonal drought, summer snow, and soil freezing continue to challenge their photochemical performance. Additionally, we observed an insignificant relation of ΦPSII with air humidity (%), PAR, and soil temperature (°C); however, with interspecific differences (Figure S13).

## 5 Conclusions

Our study revealed that the photochemical performance in high‐elevation plants varies significantly between species, with no habitat‐specific pattern. Regardless of their habitat, all species show a general decline in ΦPSII, which correlates strongly with seasonal reductions in foliar nutrient content and increased investment in leaf structure. Our findings suggest that interspecific differences in resource‐use strategies are key in shaping plant performance. Some species adopt a conservative approach, prioritising structural growth and stress tolerance, but at the expense of photochemical performance. Others manage to optimise photochemical performance while maintaining structural resilience. We show that in high‐altitude ecosystems, ΦPSII is primarily limited by SMC in moist habitats and temperature in arid habitats. However, species' responses to these environmental factors show considerable variation, potentially due to certain species‐specific adaptations. Despite their adaptations, extreme weather events, such as drought, snow, and soil freezing, impose significant stress on the plant and contribute to the decline in ΦPSII in the course of the year. These insights contribute to our understanding of plant adaptive strategies in extreme environments and emphasise the need for further investigation into how climate variability may influence high‐altitude plant function and resilience.

## Author Contributions

T.C., J.D., J.Ga., J.F., and J.Gu. designed the study and conceptualised the manuscript. T.C., J.D., and J.Ga. had the primary role in manuscript writing. T.C. performed the statistical analysis with assistance from J.D., J.B., and J.Ga. T.C. carried out the fieldwork. J.Ga. and M.J.C‐M. coordinated the laboratory analyses. All authors thoroughly reviewed and edited the manuscript.

## Supporting information


**Figure S1.** Species listed from highest to the lowest elevation of occurrence—(a) *Waldhemia tridactylites*; (b) *Primula macrophylla*; (c) *Aster flaccidus* ; (d) *Leontopodium ochroleucum*; (e) *Psychrogeton andryaloides*; (f) *Ajania fruticulosa*; (g) *Artemesia brevifolia*; (h) *Schistophyllidium bifurcum*; (i) *Potentilla pamerica*; (j) *Lactuca tatarica* ; (k) Mini‐PAM measuring at 5300 m asl; (l) In situ temperature, and soil moisture measuring data loggers.
**Figure S2.** Regression analysis of ΦPSII and *F*_*v*_/*F*_*m*_ during the growing season, represented by the day of the year. Each dot represents mean values for a species on the day of measurement, with colours distinguishing species. The *F*‐statistics and *p* values are indicated in the figure.
**Figure S3.** Mean *F*_*v*_/*F*_*m*_ with standard errors in each species from various habitats in different sampling periods. Each one‐way ANOVA performed on individual species from different habitats is independent of each other. Before performing one‐way ANOVA, assumptions were met, and if assumptions were not met, a non‐parametric Kruskal–Wallis test was used. Letters indicate significant mean differences within species, derived from a multiple comparison Tukey’s or Wilcoxon test.
**Figure S4.** Mean N content by mass in leaves with standard deviation in each species in different sampling periods. Each one‐way ANOVA performed on individual species from different habitats is independent of each other. Before performing one‐way ANOVA, assumptions were met, and if assumptions were not met, a non‐parametric Kruskal–Wallis test was used. Letters indicate significant mean differences within species, derived from a multiple comparison Tukey’s or Wilcoxon test.
**Figure S5.** Mean P content by mass in leaves with standard deviation in each species in different sampling periods. Each one‐way ANOVA performed on individual species is independent of each other. Before performing one‐way ANOVA, assumptions were met, and if assumptions were not met, a non‐parametric Kruskal–Wallis test was used. Letters indicate significant mean differences within species, derived from a multiple comparison Tukey’s or Wilcoxon test.
**Figure S6.** Mean C content by mass in leaves with standard deviation in each species in different sampling periods. Each one‐way ANOVA performed on individual species is independent of each other. Before performing one‐way ANOVA, assumptions were met, and if assumptions were not met, a non‐parametric Kruskal–Wallis test was used. Letters indicate significant mean differences within species, derived from a multiple comparison Tukey’s or Wilcoxen test.
**Figure S7.** Mean C:N ratio in leaves with standard deviation in each species in different sampling periods. Each one‐way ANOVA performed on individual species is independent of each other. Before performing one‐way ANOVA, assumptions were met, and if assumptions were not met, a non‐parametric Kruskal–Wallis test was used. Letters indicate significant mean differences within species, derived from a multiple comparison Tukey’s or Wilcoxon test.
**Figure S8.** Mean LDMC with standard deviation in each species in different sampling periods. Each one‐way ANOVA performed on individual species is independent of each other. Before performing one‐way ANOVA, assumptions were met, and if assumptions were not met, a non‐parametric Kruskal–Wallis test was used. Letters indicate significant mean differences within species, derived from a multiple comparison test.
**Figure S9.** Mean LMA with standard deviation in each species in different sampling periods. Each one‐way ANOVA performed on individual species is independent of each other. Before performing one‐way ANOVA, assumptions were met, and if assumptions were not met, a non‐parametric Kruskal–Wallis test was used. Letters indicate significant mean differences within species, derived from a multiple comparison test.
**Figure S10.** Linear regression analysis between ΦPSII and leaf traits—(a) LDMC and (b) LMA. Each line shows the regression of each species, represented by different colours with the dotted lines representing the non‐significant ones while the solid lines representing the significant results. Each dot on the plot indicates an individual.
**Figure S11.** Mean air temperature measured +6 cm with standard deviation in each species in different sampling periods. Each one‐way ANOVA performed on individual species is independent of each other. Before performing one‐way ANOVA, assumptions were met, and if assumptions were not met, a non‐parametric Kruskal–Wallis test was used. Letters indicate significant mean differences within species, derived from a multiple comparison test.
**Figure S12.** Mean soil moisture content measured −6 cm depth near the plant with standard deviation in each species in different sampling periods. Each one‐way ANOVA performed on individual species is independent of each other. Before performing one‐way ANOVA, assumptions were met, and if assumptions were not met, a non‐parametric Kruskal–Wallis test was used. Letters indicate significant mean differences within species, derived from a multiple comparison test.
**Figure S13.** Regression analysis between ΦPSII and environmental traits (air humidity and PAR)—Figure S13(a, c, e)—All species together. Each dot represents the species mean in each sampling period. Figure S13(b, d, f)—Each dots indicate an individual plant. Each species is represented by different colours. The dotted lines represent the non‐significant result while the solid lines represent the significant results.
**Table S1.** Species details—habitat of occurrence, sampling frequency, mean light‐saturating intensity used for measurement, time to achieve PSII steady‐state, and ΦPSII and leaf traits mean ± SD.
**Table S2.** Environmental data for the growing season site‐wise recorded in the year 2022–2023.

## Data Availability

All data used for this study are available in the Supporting Information.
