# Supplementary material for: Surviving the Extremes: Seasonal Dynamics of Photochemical Performance in Plants From Cold‐Arid Himalayan Mountains
Source: Physiol Plant. 2025 May 19;177(3):e70269. doi: 10.1111/ppl.70269 (PMC12087433; doi:10.1111/ppl.70269)
Supplement: Supplementary file 1 — Figure S1. Species listed from highest to the lowest elevation of occurrence—(a) Waldhemia tridactylites; (b) Primula macrophylla; (c) Aster flaccidus ; (d) Leontopodium ochroleucum; (e) Psychrogeton andryaloides; (f) Ajania fruticulosa; (g) Artemesia brevifolia; (h) Schistophyllidium bifurcum; (i) Potentilla pamerica; (j) Lactuca tatarica ; (k) Mini‐PAM measuring at 5300 m asl; (l) In situ temperature, and soil moisture measuring data loggers. Figure S2. Regression analysis of ΦPSII and F v /F m during the growing season, represented by the day of the year. Each dot represents mean values for a species on the day of measurement, with colours distinguishing species. The F‐statistics and p values are indicated in the figure. Figure S3. Mean F v /F m with standard errors in each species from various habitats in different sampling periods. Each one‐way ANOVA performed on individual species from different habitats is independent of each other. Before performing one‐way ANOVA, assumptions were met, and if assumptions were not met, a non‐parametric Kruskal–Wallis test was used. Letters indicate significant mean differences within species, derived from a multiple comparison Tukey’s or Wilcoxon test. Figure S4. Mean N content by mass in leaves with standard deviation in each species in different sampling periods. Each one‐way ANOVA performed on individual species from different habitats is independent of each other. Before performing one‐way ANOVA, assumptions were met, and if assumptions were not met, a non‐parametric Kruskal–Wallis test was used. Letters indicate significant mean differences within species, derived from a multiple comparison Tukey’s or Wilcoxon test. Figure S5. Mean P content by mass in leaves with standard deviation in each species in different sampling periods. Each one‐way ANOVA performed on individual species is independent of each other. Before performing one‐way ANOVA, assumptions were met, and if assumptions were not met, a non‐parametric Kruskal–W [file PPL-177-e70269-s001.docx]

**Surviving the Extremes: Seasonal Dynamics of Photochemical Performance in Plants from Cold-Arid Himalayan Mountains**

Thinles Chondol^1,2^, Xurxo Gago^3^, Jaume Flexas^3^, Javier Gulías^3^, María José Clemente-Moreno^3^, Jan Binter^4^, Jiří Doležal^1,2^

^1^ Department of Functional Ecology, Institute of Botany, Czech Academy of Sciences, Czech Republic

^2^ Department of Botany, Faculty of Science, University of South Bohemia, Ceske Budejovice, Czech Republic

^3^ Research Group on Plant Biology under Mediterranean Conditions, Universitat de les Illes Balears (UIB), Ctra. Valldemossa km 7.5, 07122 Palma, Spain

^4^ Department of Experimental Plant Biology, Charles University, Prague, Czech Republic

**Corresponding author:** Thinles Chondol, [thinleschondol@gmail.com](mailto:thinleschondol@gmail.com)

**Supplementary figures**


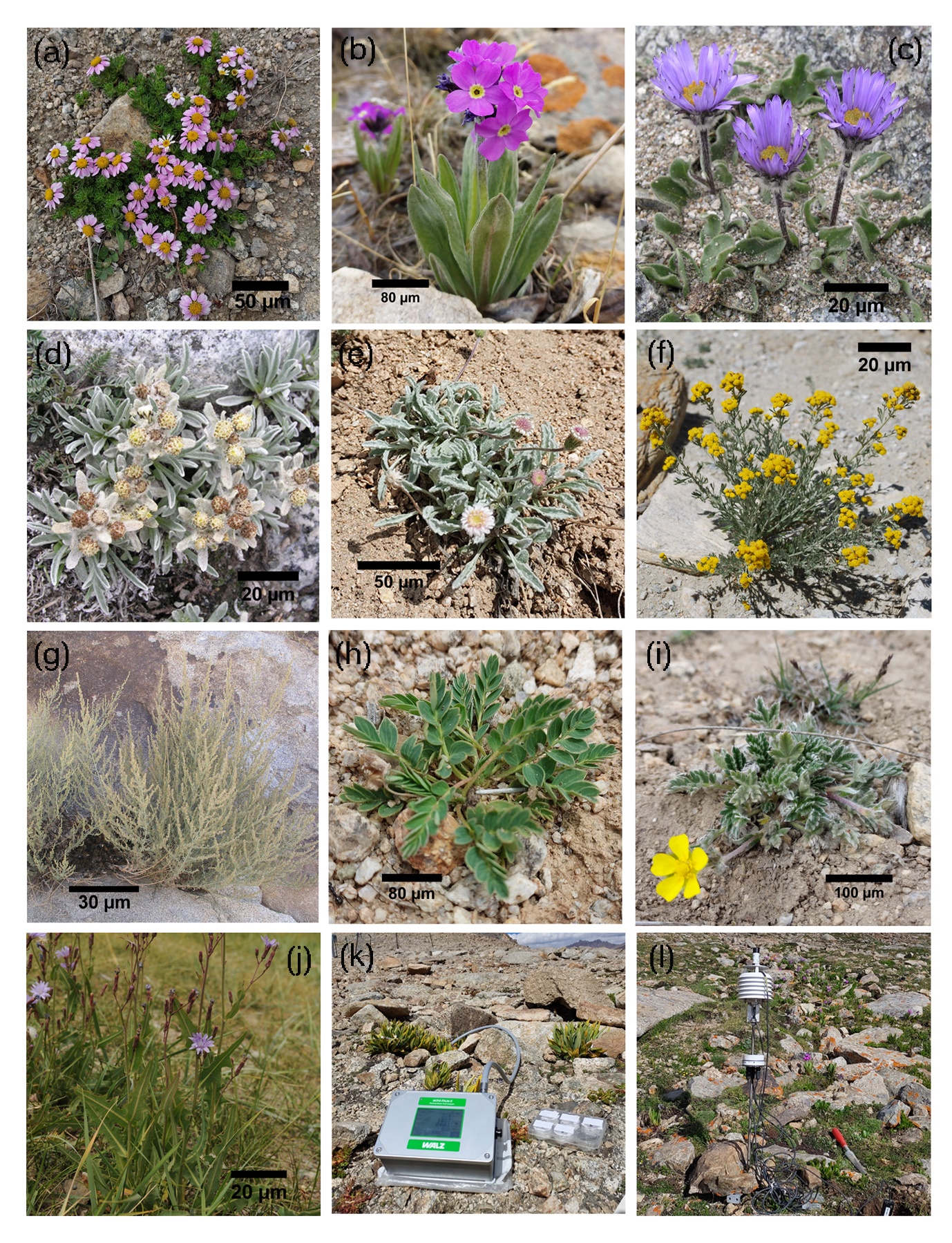


**Figure S1**: Species listed from highest to the lowest elevation of occurrence— (a) *Waldhemia tridactylites*; (b) *Primula macrophylla*; (c) *Aster flaccidus*; (d) *Leontopodium ochroleucum*; (e) *Psychrogeton andryaloides*; (f) *Ajania fruticulosa*; (g) *Artemesia brevifolia*; (h) *Schistophyllidium bifurcum*; (i) *Potentilla pamerica*; (j) *Lactuca tatarica*; (k) Mini-PAM measuring at 5300 m a.s.l.; (l) In-situ temperature, and soil moisture measuring data loggers.


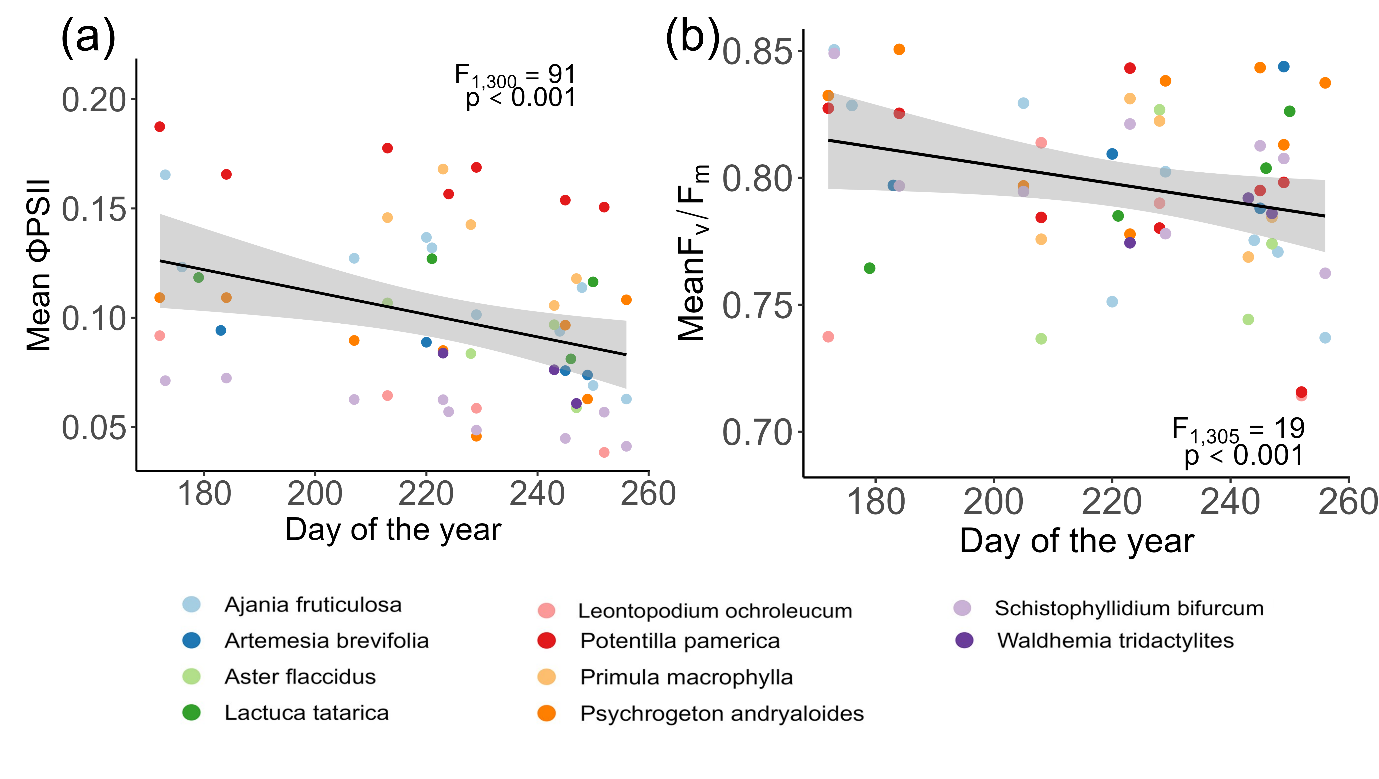


**Figure S2**: Regression analysis of ΦPSII and Fv/Fm during the growing season, represented by the day of the year. Each dot represents mean values for a species on the day of measurement, with colors distinguishing species. The F-statistics and p-values are indicated in the figure.


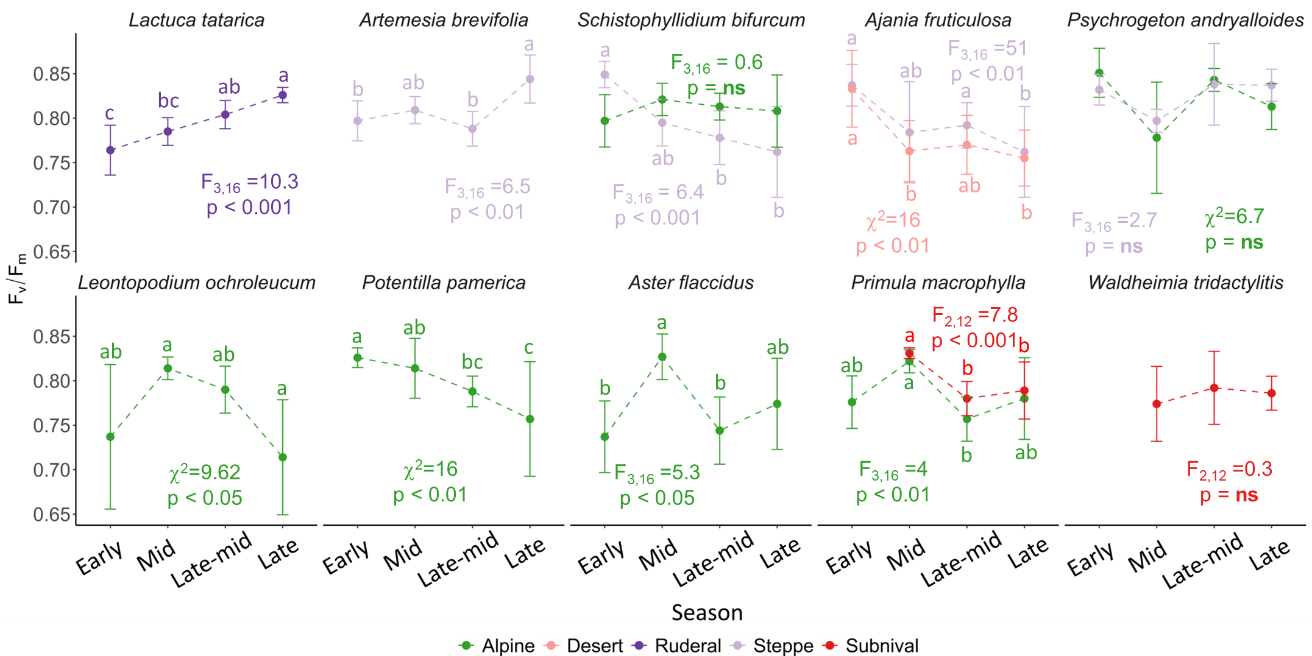


**Figure S3:** Mean F_v_/F_m_ with standard errors in each species from various habitats in different sampling periods. Each one-way ANOVA performed on individual species from different habitats is independent of each other. Before performing one-way ANOVA, assumptions were met, and if assumptions were not met, a non-parametric Kruskal-Wallis test was used. Letters indicate significant mean differences within species, derived from a multiple comparison Tukey’s or Wilcoxen test.


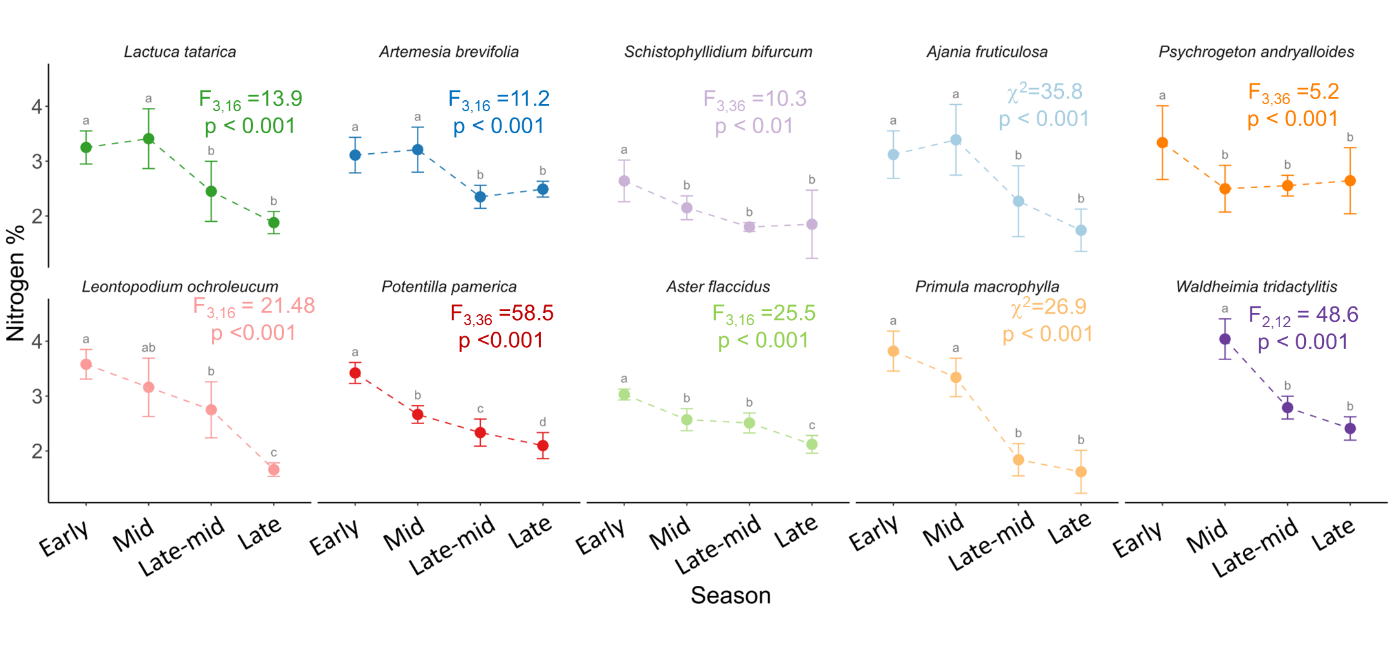


**Figure S4:** Mean N content by mass in leaves with standard deviation in each species in different sampling periods. Each one-way ANOVA performed on individual species from different habitats is independent of each other. Before performing one-way ANOVA, assumptions were met, and if assumptions were not met, a non-parametric Kruskal-Wallis test was used. Letters indicate significant mean differences within species, derived from a multiple comparison Tukey’s or Wilcoxen test.


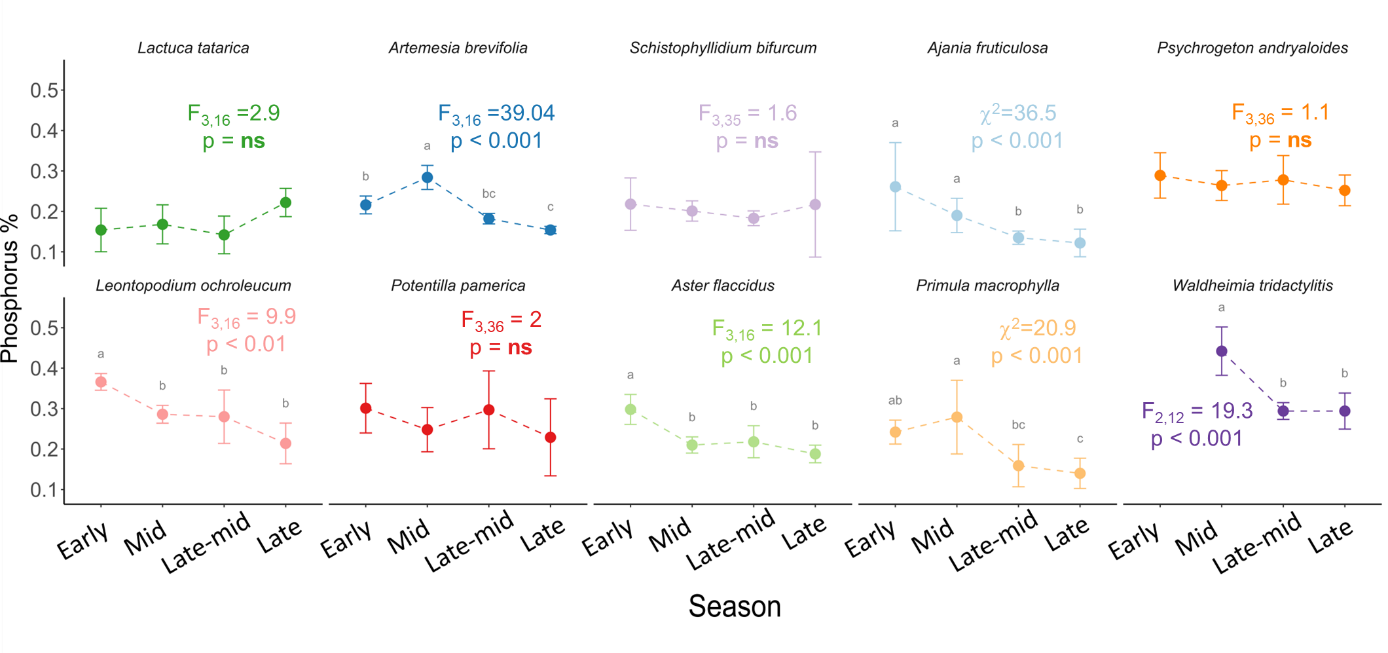


**Figure S5**: Mean P content by mass in leaves with standard deviation in each species in different sampling periods. Each one-way ANOVA performed on individual species is independent of each other. Before performing one-way ANOVA, assumptions were met, and if assumptions were not met, a non-parametric Kruskal-Wallis test was used. Letters indicate significant mean differences within species, derived from a multiple comparison Tukey’s or Wilcoxen test.


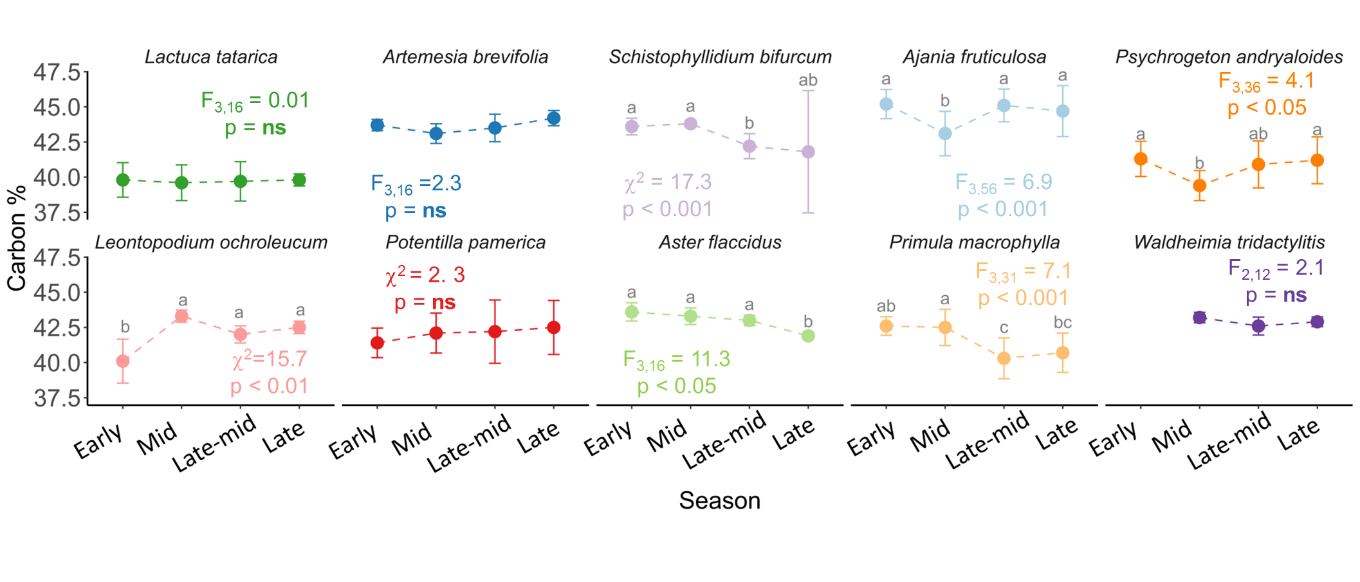


**Figure S6:** Mean C content by mass in leaves with standard deviation in each species in different sampling periods. Each one-way ANOVA performed on individual species is independent of each other. Before performing one-way ANOVA, assumptions were met, and if assumptions were not met, a non-parametric Kruskal-Wallis test was used. Letters indicate significant mean differences within species, derived from a multiple comparison Tukey’s or Wilcoxen test.


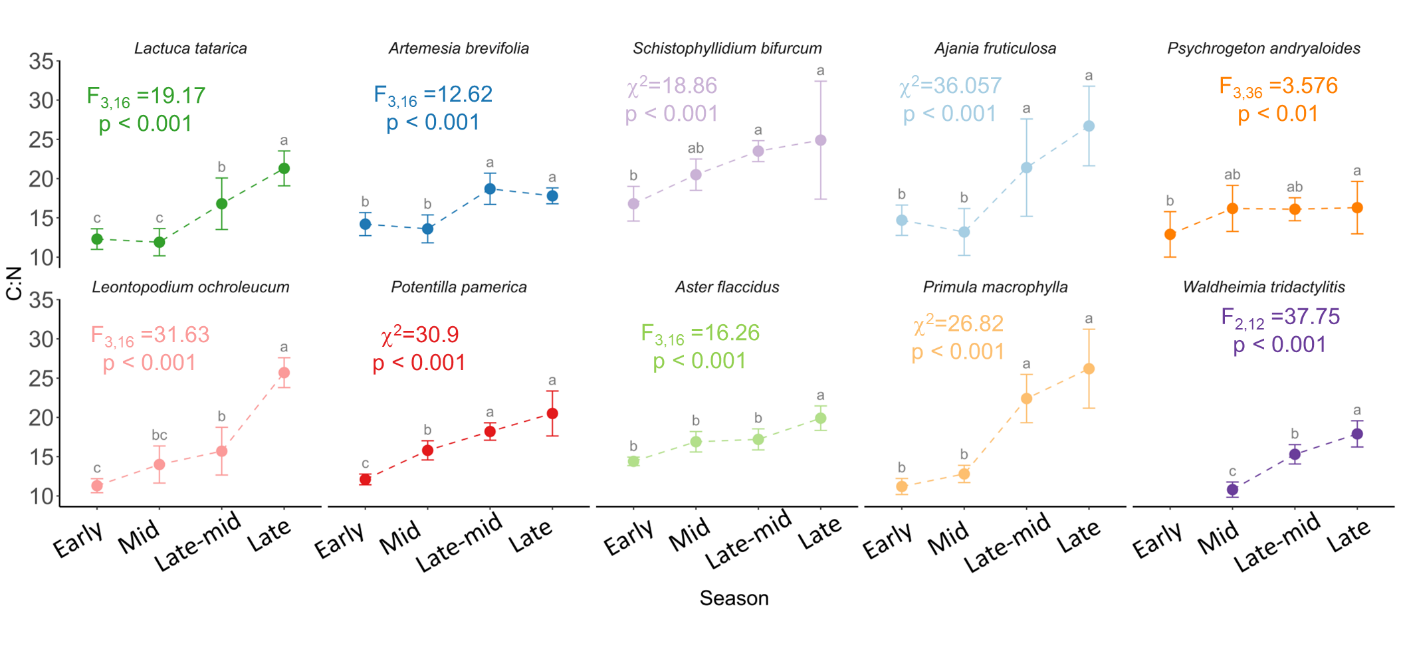


**Figure S7:** Mean C:N ratio in leaves with standard deviation in each species in different sampling periods. Each one-way ANOVA performed on individual species is independent of each other. Before performing one-way ANOVA, assumptions were met, and if assumptions were not met, a non-parametric Kruskal-Wallis test was used. Letters indicate significant mean differences within species, derived from a multiple comparison Tukey’s or Wilcoxen test.


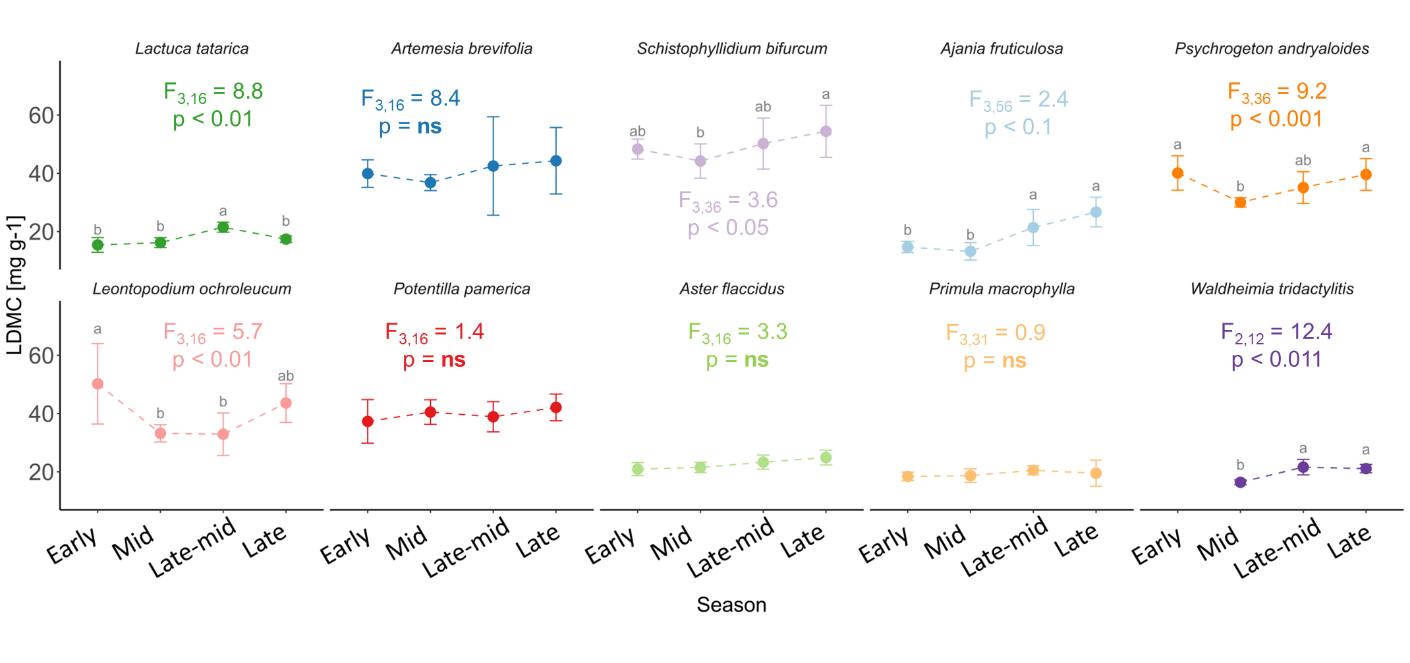


**Figure S8:** Mean LDMC with standard deviation in each species in different sampling periods. Each one-way ANOVA performed on individual species is independent of each other. Before performing one-way ANOVA, assumptions were met, and if assumptions were not met, a non-parametric Kruskal-Wallis test was used. Letters indicate significant mean differences within species, derived from a multiple comparison test.


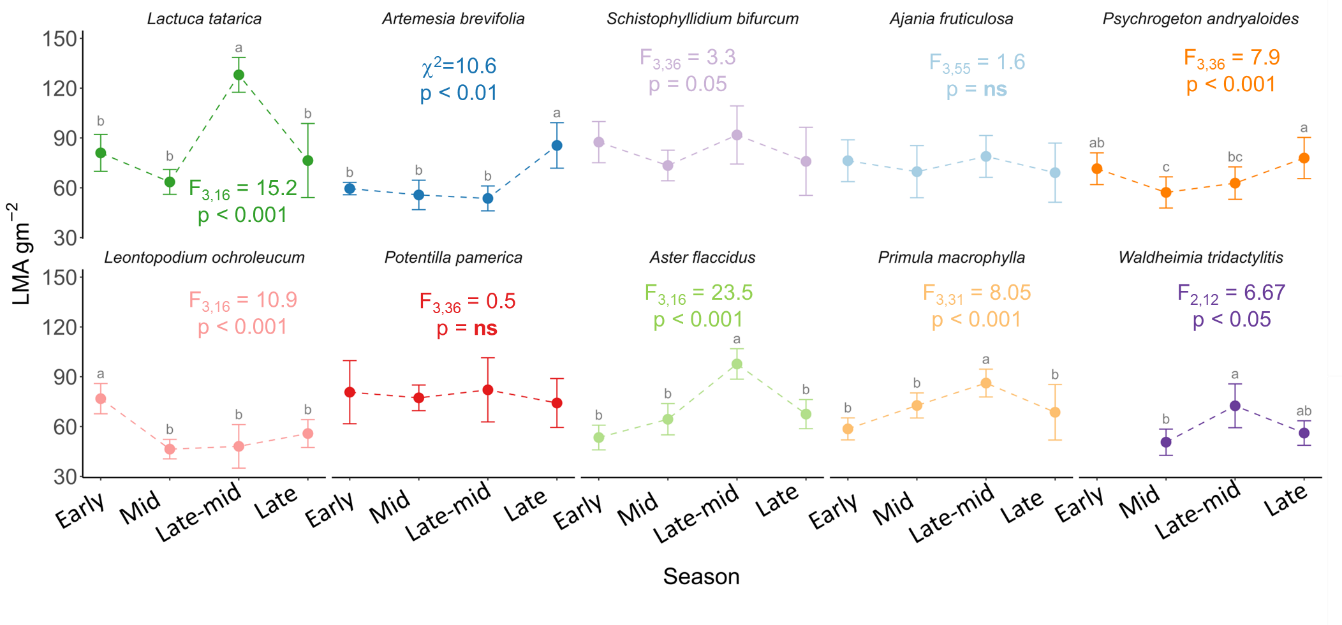


**Figure S9:** Mean LMA with standard deviation in each species in different sampling periods. Each one-way ANOVA performed on individual species is independent of each other. Before performing one-way ANOVA, assumptions were met, and if assumptions were not met, a non-parametric Kruskal-Wallis test was used. Letters indicate significant mean differences within species, derived from a multiple comparison test.


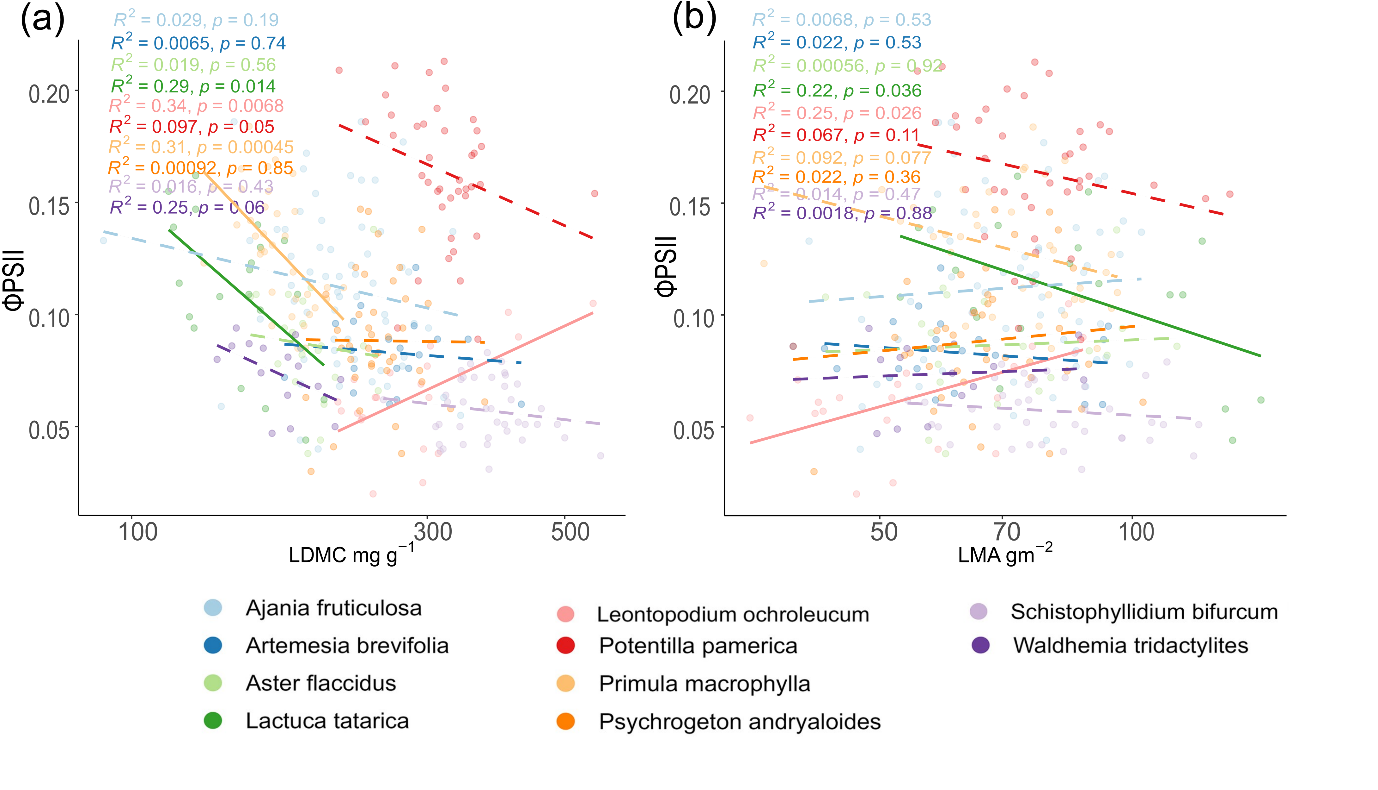


**Figure S10**: Linear regression analysis between ΦPSII and leaf traits – (a) LDMC and (b) LMA. Each line shows the regression of each species, represented by different colours with the dotted lines representing the non-significant ones while the solid lines representing the significant results. Each dot on the plot indicates an individual.


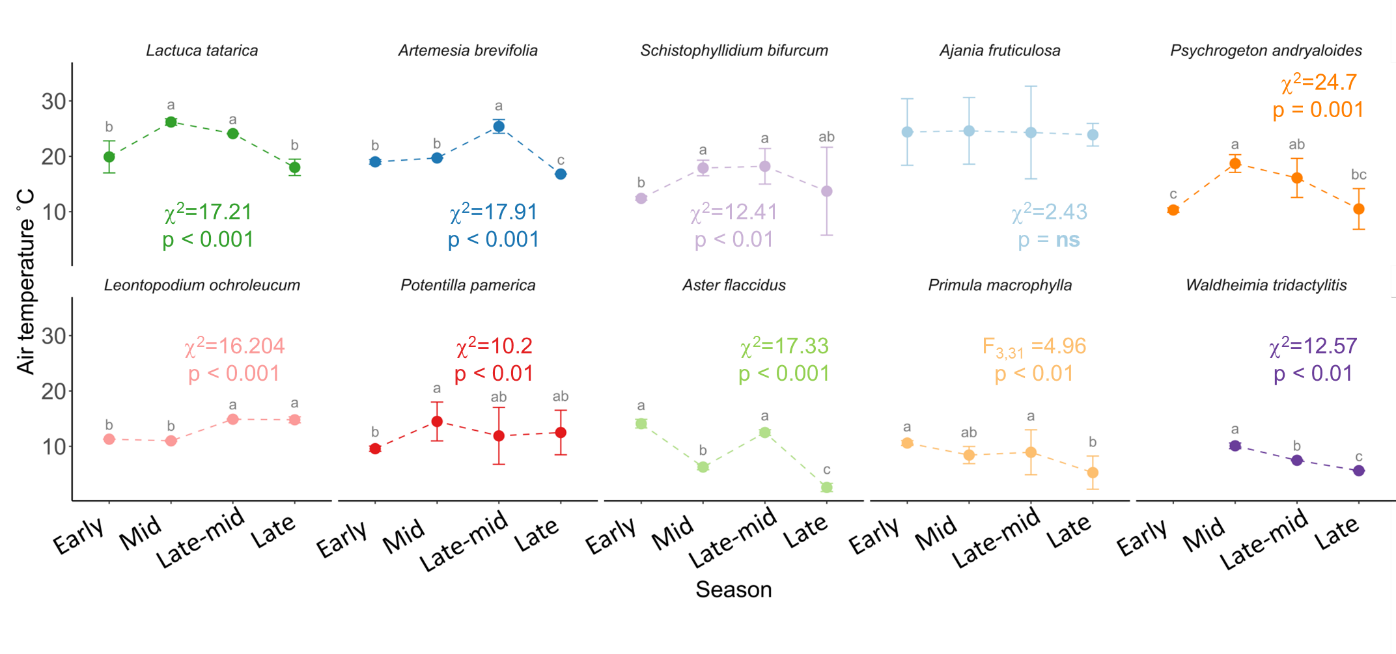


**Figure S11:** Mean air temperature measured +12 cm with standard deviation in each species in different sampling periods. Each one-way ANOVA performed on individual species is independent of each other. Before performing one-way ANOVA, assumptions were met, and if assumptions were not met, a non-parametric Kruskal-Wallis test was used. Letters indicate significant mean differences within species, derived from a multiple comparison test.


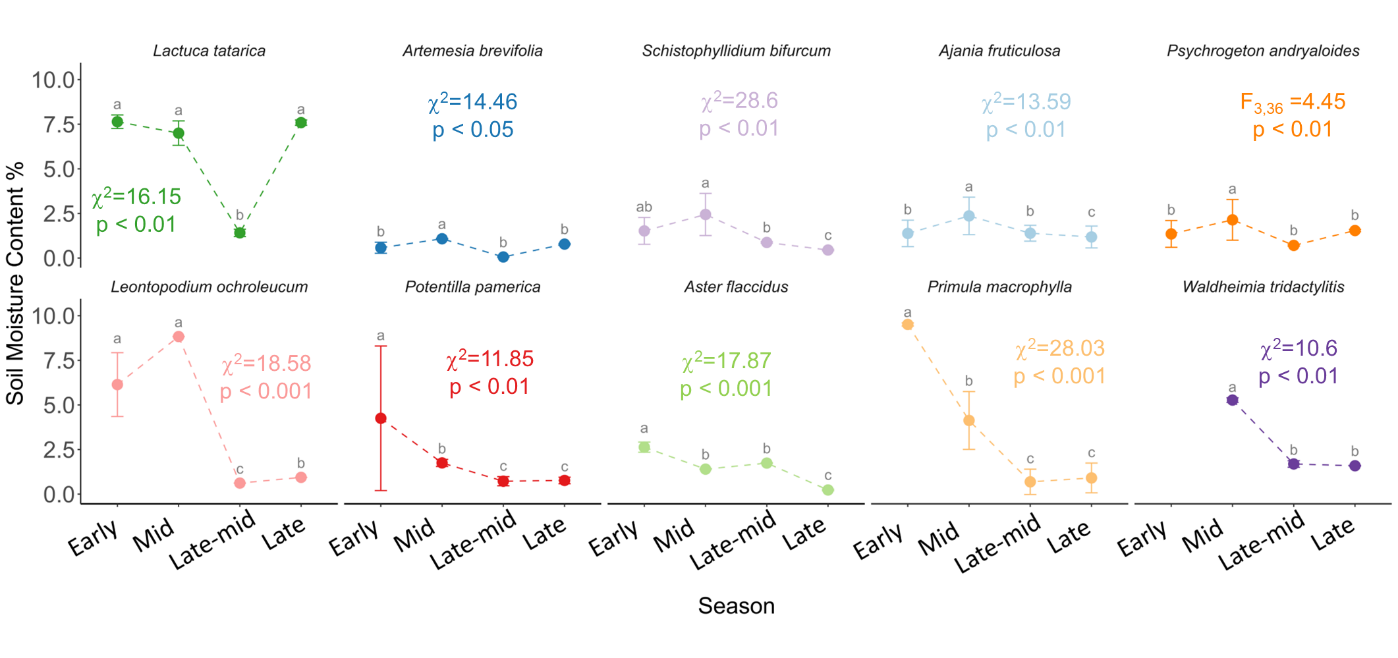


**Figure S12:** Mean soil moisture content measured near the plant with standard deviation in each species in different sampling periods. Each one-way ANOVA performed on individual species is independent of each other. Before performing one-way ANOVA, assumptions were met, and if assumptions were not met, a non-parametric Kruskal-Wallis test was used. Letters indicate significant mean differences within species, derived from a multiple comparison test.


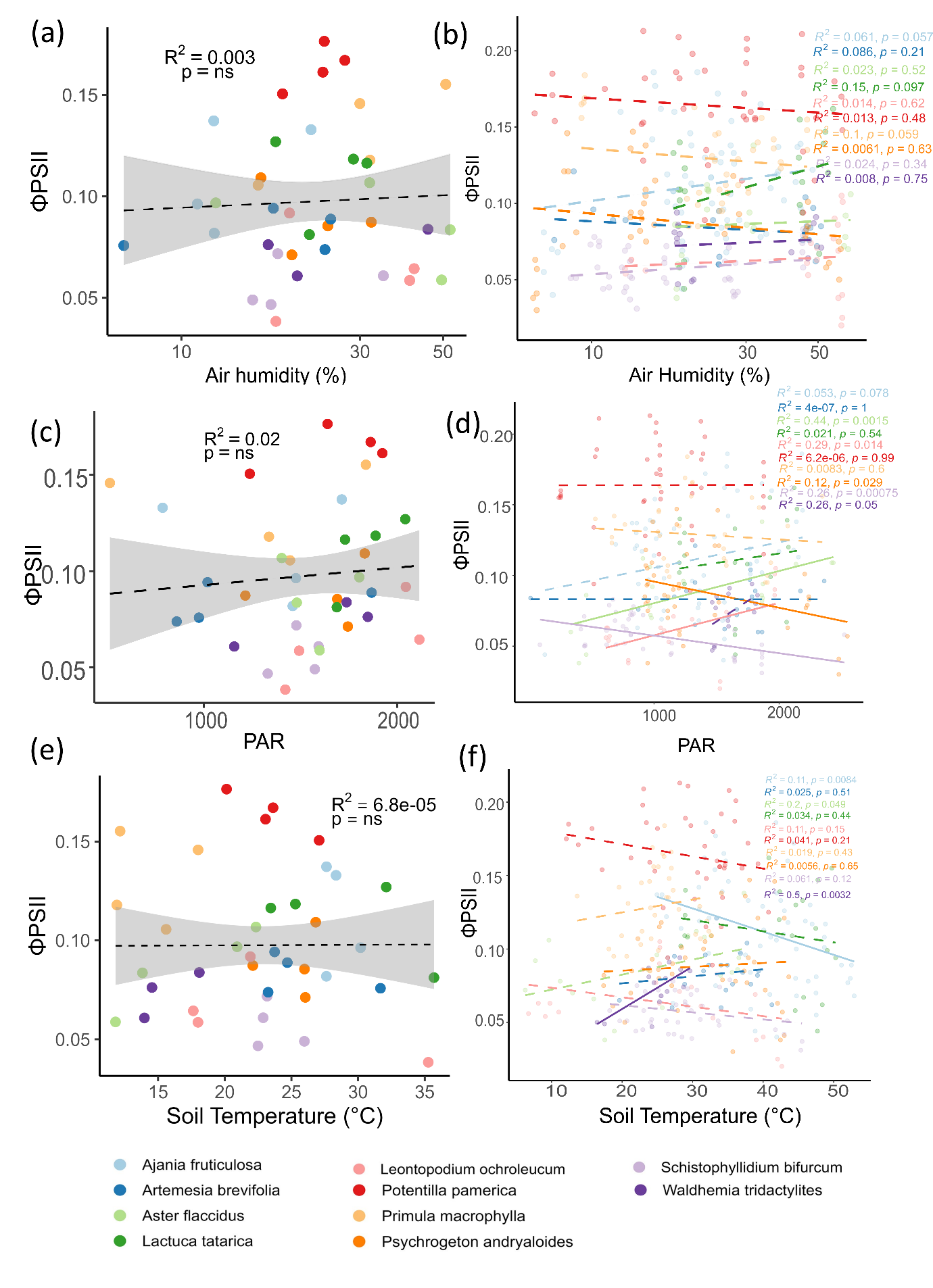


**Figure S13:** Regression analysis between ΦPSII and environmental traits (air humidity and PAR) – Figure S13(a), (c), (e) – All species together. Each dot represents the species mean in each sampling period. Figure S13(b), (d), (f) — Each dots indicate an individual plant. Each species is represented by different colours. The dotted lines represent the non-significant result while the solid lines represent the significant results.

**Table S1:** Species details – habitat of occurrence, sampling frequency, mean light-saturating intensity used for measurement, time to achieve PSII steady-state, and ΦPSII and leaf traits mean ± sd.

|  | **Habitat** | **Number of times sampled** | **Mean light intensity** | **Time (mins)** | **ΦPSIΙ** | **LDMC** | **LMA** | **N (%)** | **P (%)** | **C (%)** | **C: N** |
| --- | --- | --- | --- | --- | --- | --- | --- | --- | --- | --- | --- |
| *Ajania fruticulosa* | Steppe, semi-desert | 4 | 1676 | 15 | 0.11 ± 0.04 | 32.3 ± 9.4 | 73.4 ± 15.1 | 2.63 ± 0.85 | 0.18 ± 0.08 | 44.5 ± 1.63 | 19.01 ± 6.9 |
| *Artemesia brevifolia* | Steppe | 4 | 2332 | 16 | 0.08 ± 0.02 | 40.8 ± 10.1 | 63.6 ± 15.6 | 2.79 ± 0.47 | 0.2 ± 0.05 | 43.6 ± 0.75 | 16.05 ± 2.7 |
| *Aster flaccidus* | Alpine | 4 | 1642 | 10 | 0.09 ± 0.02 | 22.7 ± 2.6 | 70.7 ± 18.6 | 2.56 ± 0.36 | 0.22 ± 0.05 | 42.9 ± 0.8 | 17.1 ± 2.29 |
| *Lactuca tatarica* | Ruderal | 4 | 1677 | 22 | 0.11 ± 0.03 | 17.6 ± 3.1 | 87.1 ± 28.1 | 2.74 ± 0.74 | 0.17 ± 0.05 | 39.7 ± 1.05 | 15.5 ± 4.43 |
| *Leontopodium ochroleucum* | Alpine | 4 | 2315 | 12 | 0.06 ± 0.02 | 39.9 ± 10.9 | 56.7 ± 15.2 | 2.79 ± 0.82 | 0.29 ± 0.07 | 41.9 ± 1.5 | 16.7 ± 5.9 |
| *Potentilla pamerica* | Alpine | 4 | 1264 | 16 | 0.16 ± 0.03 | 39.7 ± 5.6 | 78.6 ± 15.6 | 2.63 ± 0.54 | 0.27 ± 0.08 | 42.03 ± 1.7 | 16.6 ± 3.53 |
| *Primula macrophylla* | Subnival, Alpine | 3 (subnival) 4 (alpine) | 1254 | 13 | 0.13 ± 0.03 | 19.4 ± 2.9 | 73.4 ± 14.1 | 2.49 ± 0.96 | 0.2 ± 0.08 | 41.3 ± 1.6 | 19.1 ± 6.9 |
| *Psychrogeton andryaloides* | Steppe, Low Alpine | 4 | 1824 | 15 | 0.09 ± 0.03 | 36.2 ± 6.3 | 67.3 ± 12.8 | 2.76 ± 0.6 | 0.3 ± 0.05 | 40.7 ± 1.6 | 15.36 ± 3 |
| *Schistophyllidium bifurcum* | Steppe, Low Alpine | 4 | 2317 | 16 | 0.06 ± 0.01 | 49.3 ± 7.8 | 82.1 ± 16.9 | 2.11 ± 0.5 | 0.2 ± 0.07 | 42.9 ± 2.3 | 21.4 ± 5.03 |
| *Waldhemia tridactylites* | Subnival | 3 | 2315 | 16 | 0.07 ± 0.02 | 19.7 ± 2.9 | 59.7 ± 13.3 | 3.08 ± 0.76 | 0.34 ± 0.08 | 42.9 ± 0.5 | 14.6 ± 3.3 |

**Table S2:** Environmental data for the growing season site-wise recorded in the year 2022-2023.

| **Habitat** | **Early season** | | **Mid-season** | | **Late mid-season** | | **Late season** | |
| --- | --- | --- | --- | --- | --- | --- | --- | --- |
|  | Temp_mean_  (°C) | SMC_mean_ (%) | Temp_mean_  (°C) | SMC_mean_ (%) | Temp_mean_  (°C) | SMC_mean_ (%) | Temp_mean_  (°C) | SMC_mean_ (%) |
| Subnival | -3.33 | 0.179 | 1.34 | 0.252 | 6.08 | 0.195 | 2.17 | 0.166 |
| Alpine_north-facing_ | 7.82 | 0.096 | 10.19 | 0.133 | 11.30 | 0.141 | 7.88 | 0.099 |
| Steppe_north-facing_ | 12.90 | 0.057 | 14.32 | 0.11 | 16.65 | 0.07 | 12.67 | 0.05 |
| Semi-desert | 19.70 | 0.08 | 20.91 | 0.10 | 23.63 | 0.09 | 19.24 | 0.08 |
| Alpine_south-facing_ | -3.32 | 0.14 | 1.34 | 0.13 | 6.13 | 0.15 | 2.17 | 0.11 |
| Steppe_south-facing_ | 15.42 | 0.05 | 16.98 | 0.155 | 19.02 | 0.08 | 17.55 | 0.05 |
